# Piezo1-Mediated Ferroptosis Delays Wound Healing in Aging Mice by Regulating the Transcriptional Activity of SLC7A11 through Activating Transcription Factor 3

**DOI:** 10.34133/research.0718

**Published:** 2025-06-03

**Authors:** Chen Jin, Du-piao Zhang, Zhen Lin, Yu-zhe Lin, Yi-feng Shi, Xiao-yu Dong, Meng-qi Jin, Fu-qiang Song, Si-ting Du, Yan-zhen Feng, Lin-yuan Jiang, Xiao-qiong Jiang, Abdullah Al Mamun, Zi-miao Chen, Jian Wang, Keqing Shi, Ren-wen Wan, Zhi-wen Luo, Zheng-lin Li, Lei Yang, Jian Xiao

**Affiliations:** ^1^ Department of Wound Healing, The First Affiliated Hospital of Wenzhou Medical University, Wenzhou 325015, China.; ^2^ Oujiang Laboratory (Zhejiang Lab for Regenerative Medicine, Vision and Brain Health), School of Pharmaceutical Sciences, Wenzhou Medical University, Wenzhou, Zhejiang 325035, China.; ^3^ Department of Orthopaedics, Zhejiang Provincial Key Laboratory of Orthopaedics, The Second Affiliated Hospital and Yuying Children’s Hospital of Wenzhou Medical University, Wenzhou 325027, China.; ^4^ Nanfang Hospital, Southern Medical University, Guangzhou 510515, China.; ^5^ Central Laboratory of The Lishui Hospital of Wenzhou Medical University, The First Affiliated Hospital of Lishui University, Lishui People’s Hospital, Lishui, Zhejiang 323000, China.; ^6^ Department of Sports Medicine, Huashan Hospital, Fudan University, Shanghai 200000, China.

## Abstract

Ferroptosis plays a role in wound healing during the maturation of senescent endothelial cells. This study explores the modulation of ferroptosis in senescent human umbilical vein endothelial cells (HUVECs) and wound-healing processes by Piezo1 activation at the molecular, cellular, and tissue levels. Elevated Piezo1 expression was observed in HUVECs treated with the senescence inducer doxorubicin (Doxo) and the ferroptosis inducer erastin and in aged wound tissue. Pharmacological inhibition or knockdown of Piezo1 protected senescent HUVECs and aged wound tissue from ferroptosis. Additionally, Piezo1 channel activity was found to promote ferroptosis in senescent HUVECs by increasing intracellular Ca^2+^ levels. The calmodulin-dependent kinase II (CaMKII)/activating transcription factor 3 (ATF3)/SLC7A11 signaling axis was activated upon stimulation with erastin and Doxo, driving Piezo1-induced ferroptosis. CaMKII directly interacted with ATF3, which could be modulated through Piezo1 channel regulation. Notably, Piezo1 knockout mice or adeno-associated virus 9-mediated silencing of ATF3 attenuated ferroptosis in senescent cells and accelerated wound repair. Mechanistically, both genetic and pharmacological inhibition of Piezo1 promoted wound healing in aged tissues and regulated ferroptosis in senescent HUVECs through the CaMKII/ATF3/SLC7A11 pathway. In conclusion, these findings suggest that targeting Piezo1-mediated ferroptosis in senescent HUVECs offers a promising therapeutic approach for improving wound healing in the elderly.

## Introduction

The accumulation of senescent cells in various tissues during natural aging significantly impairs the body’s capacity to repair and maintain tissues [1]. Cellular senescence is a critical biological mechanism underlying age-related dysfunctions and chronic diseases, particularly hindering geriatric wound healing [2]. Delayed wound healing imposes substantial economic burdens on healthcare systems, with chronic wound care costs exceeding $25 billion annually, while also profoundly impacting patients’ quality of life [3]. The delicate and vulnerable skin of older individuals makes them more prone to bruising and delayed wound healing [4]. Several factors contributing to the impaired healing of senescent human umbilical vein endothelial cells (HUVECs) have been identified, primarily stemming from disruptions within the intracellular environment. Numerous studies have emphasized the pivotal role of damage-induced senescence in the wound-healing process [5,6]. However, the precise mechanisms governing age-related wound healing remain poorly understood.

Ferroptosis, a form of programmed cell death driven by iron-dependent oxidative damage, lipid peroxidation, and reactive oxygen species (ROS) accumulation, plays a critical role in cellular dysfunction [7,8]. These processes impair glutathione (GSH) synthesis and inhibit glutathione peroxidase 4 (GPX4), an enzyme dependent on GSH for its activity. Excessive ROS production, a hallmark of oxidative stress, induces mitochondrial dysfunction, exacerbates lipid peroxidation, and ultimately triggers ferroptosis. Inhibition of ferroptosis has been shown to reduce intracellular lipid peroxidation, promote cell proliferation, and restore cellular function, suggesting that ferroptosis may be integral to wound healing [9,10]. However, the exact role and regulatory mechanisms of ferroptosis in senescent HUVECs remain to be elucidated.

Calcium ions (Ca^2+^) serve as critical second messengers in various cellular processes, including proliferation, apoptosis, and autophagy [11]. Their accumulation is closely linked to cellular senescence [12]. Recent studies have demonstrated that elevated intracellular Ca^2+^ levels significantly promote senescence, primarily through mechanisms involving mitochondrial dysfunction and ROS production [13,14]. Additionally, it has been demonstrated that excessive calcium influx and heightened calpain activity contribute to ferroptosis in endothelial cells [15,16]. Calmodulin-dependent kinase II (CaMKII), a downstream kinase activated by Ca^2+^ influx, plays a pivotal role in wound repair and skin development [17,18]. Given that Piezo1 activation induces a transient influx of Ca^2+^, it is reasonable to propose that CaMKII mediates the downstream signaling of Piezo1[19]. The Piezo1 ion channel, abundantly expressed in the skin, facilitates Ca^2+^ entry into cells, a key step in accelerating wound healing, underscoring its importance in tissue repair and regeneration [16,20]. However, excessive Piezo1 activation under pathological stress may lead to dysfunction in HUVECs. Hirata et al. reported that elevated Piezo1 expression accelerates membrane lipid oxidation, leading to increased intracellular Ca^2+^ levels. This up-regulation serves as a key event in modulating cellular functions and signaling pathways. Piezo1 regulates Ca^2+^ release in senescent HUVECs, likely by promoting Ca^2+^ influx or mobilizing intracellular stores. Activating transcription factor 3 (ATF3), a member of the ATF/adenosine 3′,5′-monophosphate response element-binding protein (CREB) family, is regulated by Ca^2+^ influx and has been implicated in ferroptosis regulation [21]. Kang et al. [22] showed that calcium influx enhances ATF3 expression and lipid peroxidation, while Li et al. [23] established a strong link between calcium influx and ATF3 signaling in the aging process of mesenchymal stem cells. Thus, it is hypothesized that Piezo1-mediated Ca^2+^ overload could represent a novel therapeutic target for treating ferroptosis-related diseases [24,25]. Our previous research highlighted the elevated expression of both ferroptosis markers and Piezo1 at wound sites in aging mice, with Piezo1 inhibition accelerating wound healing. However, the precise role of Piezo1 in mitigating ferroptosis in senescent HUVECs, along with the underlying molecular mechanisms, remains poorly understood.

This study examined wound samples from aged patients and mice to investigate the potential role of ferroptosis in wound healing. Additionally, the molecular mechanisms through which Piezo1 influences doxorubicin (Doxo)-induced ferroptosis in HUVECs were elucidated through both Piezo1 knockdown and pharmacological inhibition. To assess the impact of Piezo1 and ATF3 on wound healing and ferroptosis in vivo, aging mice were treated with cKO-Piezo1 mice and adeno-associated virus-mediated short hairpin RNA (shRNA) targeting ATF3. Based on our experimental results, targeting and inhibiting Piezo1-mediated ferroptosis emerges as a promising therapeutic strategy for enhancing wound repair in aging tissues, offering a novel treatment approach.

## Results

### Increased ferroptosis and abnormal accumulation of Piezo1 in aged wounds both in vitro and in vivo

**Fig. 1.**
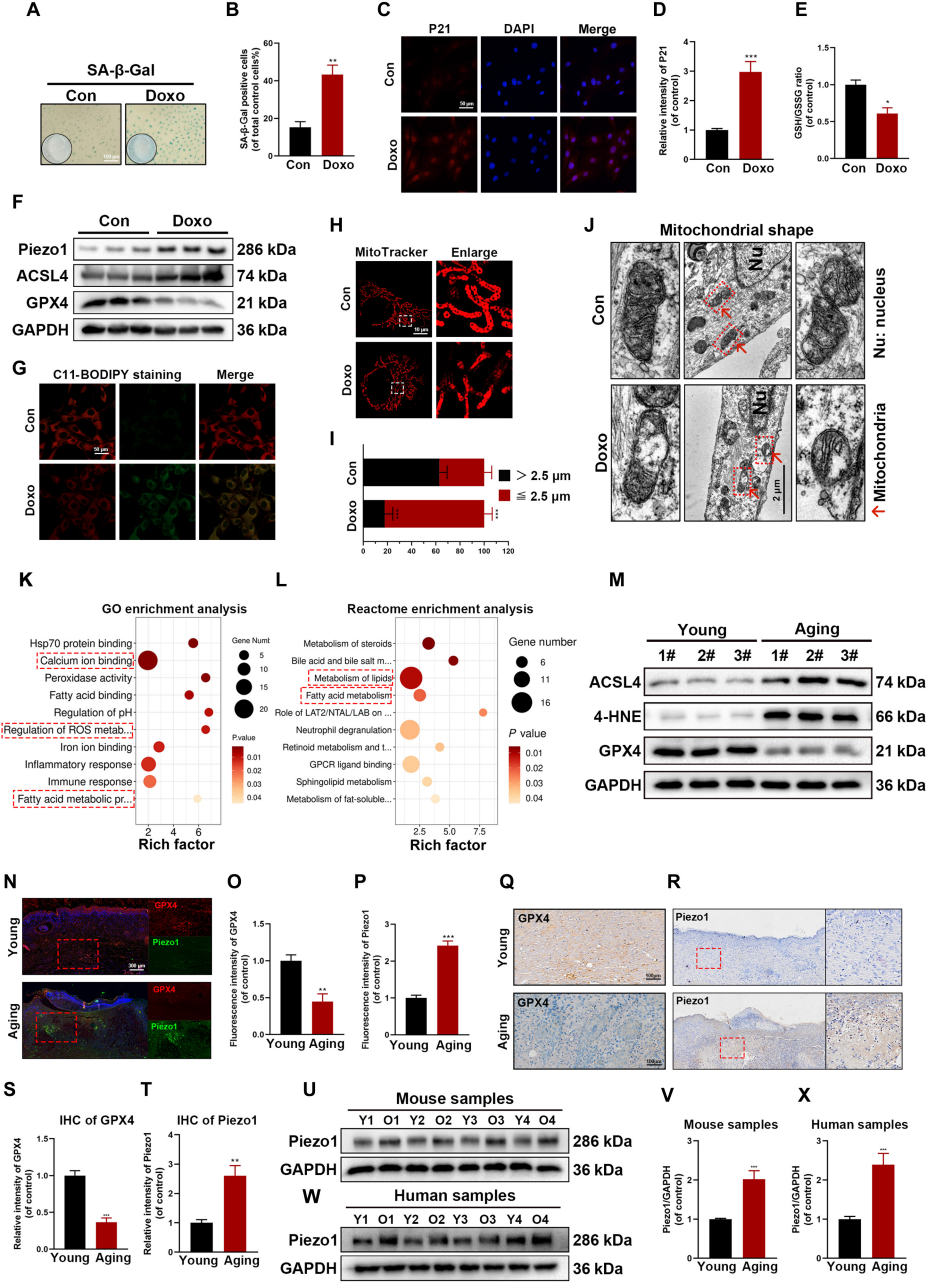
Increased ferroptosis and abnormal accumulation of Piezo1 in aged wounds in vitro and in vivo. (A and B) SA-β-Gal expression and quantification analysis in HUVECs treated with/without Doxo. (C and D) Representative IF images and quantification analysis of p21 in HUVECs treated with/without Doxo. (E) GSH/GSSG ratio was measured in HUVECs treated with/without Doxo. (F) Western blot analysis for ferroptosis biomarkers ACSL4, GPX4, and Piezo1 in HUVECs treated with/without Doxo. (G to J) Representative images of C11-BODIPY staining and MitoTracker staining (red) and TEM images. (K and L) GO and Reactome enrichment analyses show fatty acid metabolism and metabolism of lipids and ROS associated with these DEGs. (M) Western blot analysis for ferroptosis biomarkers ACSL4, 4-HNE, and GPX4 in young and aging groups in mouse wound tissue. (N to P) Representative IF images and quantification analysis of GPX4 and Piezo1 in young and aging groups in mouse wound tissue. (Q to T) Representative IHC images and quantification analysis of GPX4 and Piezo1 in young and aging groups in mouse wound tissue. (U to X) Western blot analysis of Piezo1 in young and aging groups of mouse samples and human samples. * indicates a comparison between the 2 groups. *P < 0.05; **P < 0.01; ***P < 0.001. All data are performed in triplicate and at least 3 times.

Previous research has shown that Doxo induces cellular aging [26]. Senescence phenotypes were assessed using the senescence-β-galactosidase (SA-β-Gal) staining kit, and HUVECs treated with Doxo (100 nM) for 48 h exhibited increased SA-β-Gal expression (Fig. 1A and B). As a member of the senescence-associated protein family, p21 expression was also elevated in Doxo-treated HUVECs, as confirmed by immunofluorescence (IF) staining (Fig. 1C and D). Therefore, a concentration of 100 nM Doxo was used to induce senescence in HUVECs for subsequent experiments. Reduced GSH is a critical component of the defense system against ferroptosis, and the GSH/GSSG (oxidized glutathione) ratio serves as an indicator of ferroptosis levels. The GSH/GSSG assay kit was employed to measure the levels of GSH and oxidized GSH in vitro. Doxo treatment significantly decreased the GSH/GSSG ratio (Fig. 1E). Additionally, Western blot analysis revealed that Doxo treatment up-regulated ACSL4 expression and down-regulated GPX4 expression, suggesting that Doxo may contribute to ferroptosis (Fig. 1F and Fig. S1A). C11-BODIPY and FerroOrange probes were used to directly assess intracellular lipid peroxidation and free ferrous ion levels. Doxo treatment resulted in significant lipid peroxidation and elevated iron levels, as evidenced by C11-BODIPY and FerroOrange staining (Fig. 1G and Fig. S1B to D). Mitochondrial damage, another hallmark of ferroptosis, was observed, which is widely regarded as a key indicator of ferroptosis. Both IF and transmission electron microscopy (TEM) images showed that Doxo-treated cells exhibited smaller, swollen mitochondria, large vacuoles, and ruptured outer mitochondrial membranes (Fig. 1H to J) [27]. To explore the underlying mechanisms, RNA sequencing was conducted on young (10-week-old mice) and aging (18-month-old mice) wound groups to investigate the molecular processes involved in wound aging. Differentially expressed genes (DEGs) were visualized using a volcano plot, which revealed significant alterations in 813 genes—311 down-regulated and 502 up-regulated (Fig. S1E). Gene Ontology (GO) analysis indicated that DEGs were primarily associated with fatty acid metabolism, ROS metabolism, and calcium binding (Fig. 1K). Reactome enrichment analysis highlighted that fatty acid and lipid metabolism pathways were notably enriched in the aging group (Fig. 1L). Gene Set Enrichment Analysis (GSEA) further revealed a significant reduction in GSH transferase activity and GSH metabolism in the aging wound groups (Fig. S1F). Moreover, data from GSE262932 suggest that Ca^2+^ signaling pathway, lipid metabolic process, and fatty acid metabolic process are enriched in aging human skin (Fig. S3A and B). These findings suggest that ferroptosis may play a pivotal role in the progression of aging-related wound healing. This study also examined key ferroptosis regulators and found that the expression of ACSL4 and 4-hydroxynonenal (4-HNE) was significantly increased, while GPX4 expression was markedly decreased in aging wound groups (Fig. 1M and Fig. S1G). Immunohistochemical (IHC) and IF staining confirmed that GPX4 expression was reduced in aging wound groups (Fig. 1N, O, Q, and S). Furthermore, Piezo1 expression was significantly elevated in both senescent HUVECs and aging wounds (Fig. 1F, U, and V), IHC and IF staining also confirmed (Fig. 1N, P, R, and T). Interestingly, a similar pattern of increased Piezo1 expression was observed in wounds from elderly patients (Fig. 1W and X). Taken together, these results indicate that ferroptosis and the abnormal accumulation of Piezo1 are heightened in senescent HUVECs and aging wounds.

### Inhibition of ferroptosis promotes the healing of aging wounds in vivo

Fer-1, a well-established inhibitor of ferroptosis, was utilized to explore the potential of inhibiting ferroptosis in aging tissues to enhance wound healing [9]. To evaluate the role of Fer-1 in aging-related wound healing, aging models were treated with Fer-1 (2 μg/kg, dissolved in 0.01% dimethyl sulfoxide) via wound base injections. The mice were divided into 3 groups (*n* = 8): Young (10-week-old mice), Aging (18-month-old mice), and Aging + Fer-1 (18-month-old mice with Fer-1 treatment). Gross morphological changes were observed at days 0, 3, and 7 post-surgery. Over time, all groups (Young, Aging, and Aging + Fer-1) demonstrated a progressive reduction in wound area; however, the Aging + Fer-1 group exhibited a significantly greater reduction compared to the Aging group at both 3 and 7 d (Fig. S2A and B). Histological analyses, including hematoxylin and eosin (H&E) and Masson’s trichrome staining, were performed to assess key pathological features of wound healing, such as reepithelialization, granulation tissue formation, and wound length. Quantitative analysis of H&E-stained sections revealed that the Aging + Fer-1 group had a significantly shorter wound length than the Aging group (Fig. S2C, D, and G). Additionally, IHC staining for vascular endothelial growth factor-A (VEGF-A), an angiogenesis marker, showed that the Aging + Fer-1 group had higher VEGF-A expression compared to the Aging group, corroborating the H&E results (Fig. S2E and H). Further investigation revealed that Fer-1 treatment reduced the expression levels of 4-HNE, a secondary aldehyde product of lipid peroxidation (Fig. S2F and I). Taken together, these results suggest that inhibition of ferroptosis significantly accelerates wound healing in aging tissues.

### Pharmacological inhibition of Piezo1 alleviates ferroptosis in senescent HUVECs

**Fig. 2.**
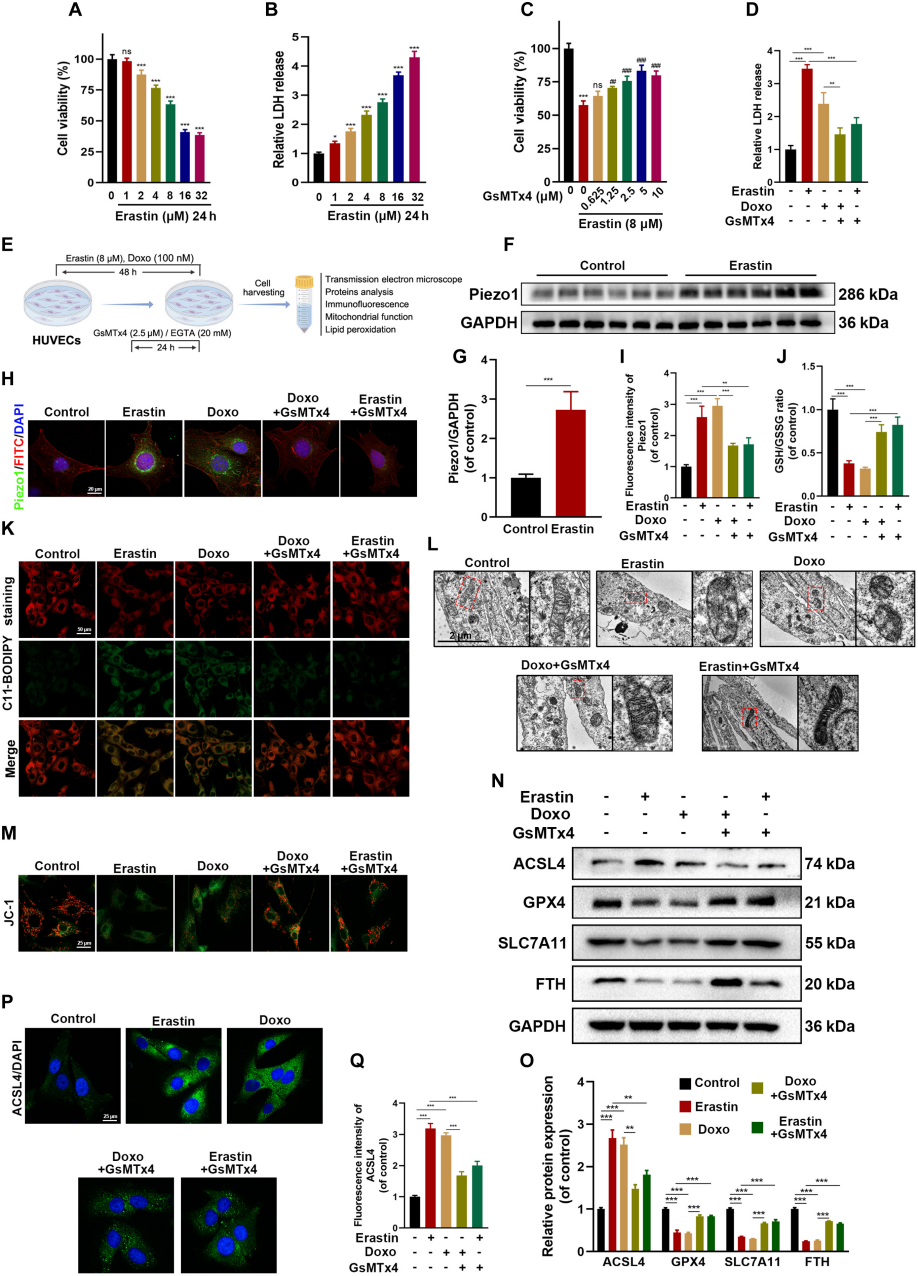
Inhibition of ferroptosis promotes the healing of aging wounds in vivo. (A) Cell viability of HUVECs treated with different concentrations of erastin for 24 h. (B) LDH release of HUVECs treated with different concentrations of erastin for 24 h. (C) Cell viability of HUVECs treated with different concentrations of GsMTx4 with/without erastin for 24 h. (D) LDH release of HUVECs with different treatments for 24 h. (E) Schematic illustration of vitro experiment. (F and G) Western blot analysis for Piezo1 in HUVECs with/without erastin. (H and I) Representative IF images and quantification analysis of Piezo1 in HUVECs with different treatments. (J) GSH/GSSG ratio was measured in HUVECs with different treatments. (K to M) Representative images of C11-BODIPY staining, TEM, and JC-1 staining in HUVECs with different treatments. (N and O) Western blot analysis for ferroptosis biomarkers ACSL4, SLC7A11, FTH, and GPX4 in HUVECs with different treatments. (P and Q) Representative IF images and quantification analysis of ACSL4 in HUVECs with different treatments. *P < 0.05; **P < 0.01; ***P < 0.001. All data are performed in triplicate and at least 3 times.

Ferroptosis was induced in vitro using the agent erastin to investigate the involvement of Piezo1 in Doxo-treated HUVEC ferroptosis, as illustrated in Fig. 2E. Erastin treatment significantly reduced cell viability in a concentration-dependent manner and up-regulated Piezo1 expression in HUVECs at 8 μM, suggesting Piezo1’s pivotal role in ferroptosis progression (Fig. 2A, F, and G). Additionally, lactate dehydrogenase (LDH) activity was significantly elevated following 24-h erastin exposure in a concentration-dependent fashion (Fig. 2B). Notably, GsMTx4 (Piezo1 inhibitor) attenuated erastin- and Doxo-induced HUVEC death, decreased LDH activity, and improved the GSH/GSSG ratio (Fig. 2C and D). IF images confirmed that GsMTx4 suppressed Piezo1 expression in HUVECs exposed to either Doxo or erastin (Fig. 2H and I). Moreover, 5-ethynyl-20-deoxyuridine (EdU) staining indicated that GsMTx4 enhanced cell proliferation (Fig. S3F and H). Ferroptosis, marked by lipid peroxidation, iron accumulation, and mitochondrial dysfunction, was further assessed using C11-BODIPY and FerroOrange staining, alongside TEM, in HUVECs treated with erastin and Doxo, with or without GsMTx4. C11-BODIPY staining showed significant lipid peroxidation upon erastin and Doxo treatment, which was alleviated by GsMTx4 (Fig. 2K and Fig. S3C). FerroOrange staining revealed a marked increase in fluorescence intensity upon erastin and Doxo exposure, which was reversed by GsMTx4 (Fig. S3E and G). TEM analysis identified classic ferroptosis hallmarks, including mitochondrial shrinkage and increased membrane density, both of which were substantially ameliorated by GsMTx4 treatment (Fig. 2L). GsMTx4 also restored mitochondrial membrane potential (MMP) defects, consistent with TEM findings (Fig. 2M and Fig. S3D). Additionally, differential expression of ferroptosis core regulators was observed in HUVECs treated with erastin and Doxo. Specifically, ACSL4 was up-regulated, while SLC7A11, FTH, and GPX4 were down-regulated. GsMTx4 treatment reversed these changes, as shown in Fig. 2N and O. IF imaging further confirmed GsMTx4’s reduction of ACSL4 expression, as depicted in Fig. 2P and Q. These results collectively highlight the effectiveness of Piezo1 inhibition in mitigating erastin- and Doxo-induced ferroptosis in senescent HUVECs.

### Inhibition of the Piezo1 channel alleviates ferroptosis in HUVECs via a Ca^2+^-dependent mechanism

**Fig. 3.**
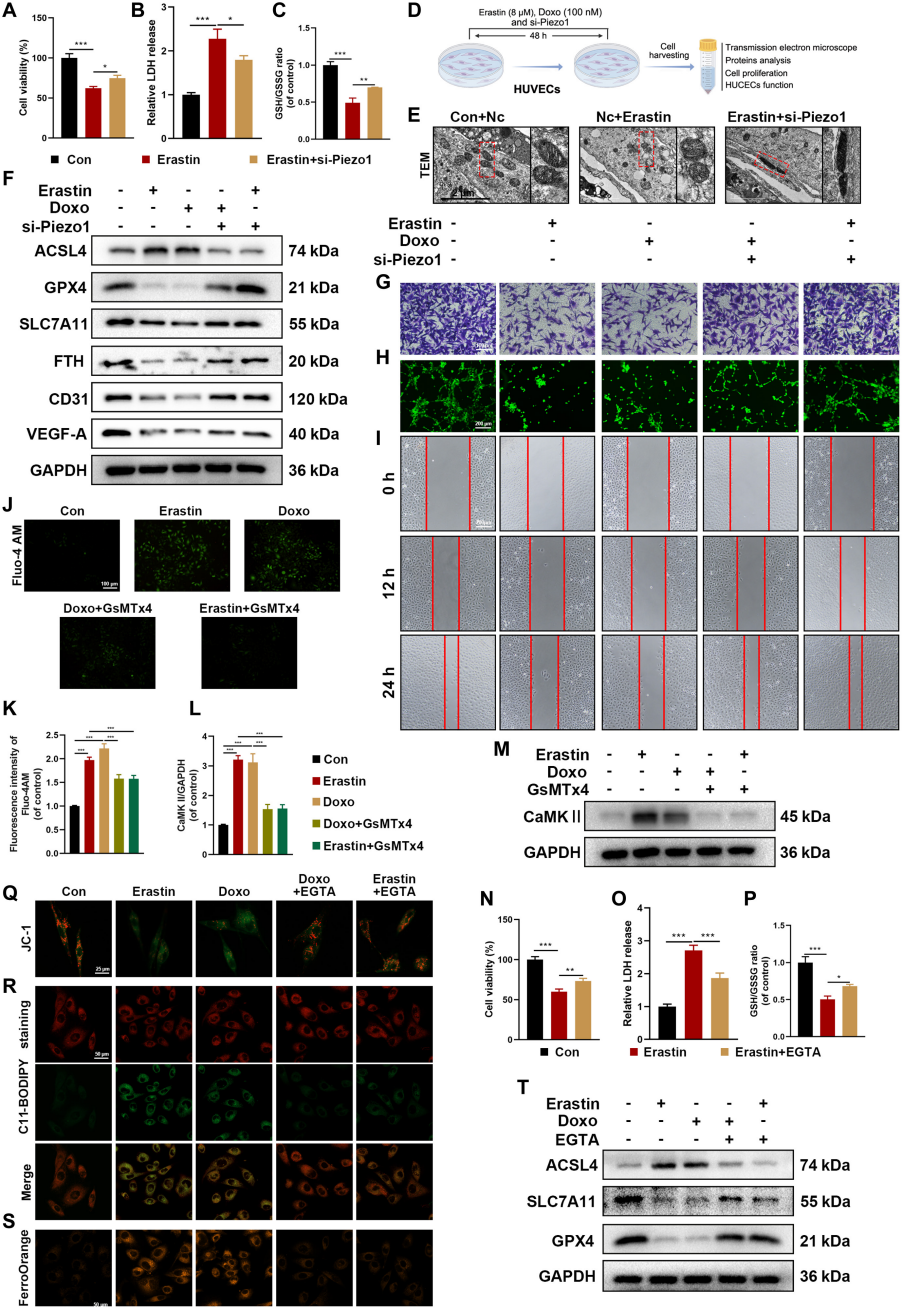
Inhibition of Piezo1 alleviates ferroptosis in senescent HUVECs. (A) Cell viability of HUVECs with different treatments for 24 h. (B) LDH release of HUVECs with different treatments for 24 h. (C) GSH/GSSG ratio was measured in HUVECs with different treatments. (D) Schematic illustration of vitro experiment. (E) Representative images of TEM with different treatments. (F) Western blot analysis for ferroptosis and angiogenesis biomarkers ACLS4, GPX4, SLC7A11, FTH, CD31, and VEGF-A in HUVECs with different treatments. (G to I) Representative images of migration assay, tube formation assay, and scratch experiment in HUVECs with different treatments. (J and K) Representative images and quantification analysis of Fluo-4 AM staining in HUVECs with different treatments. (L and M) Western blot analysis for CaMKII in HUVECs with different treatments. (N) Cell viability of HUVECs with different treatments for 24 h. (O) LDH release of HUVECs with different treatments for 24 h. (P) GSH/GSSG ratio was measured in HUVECs with different treatments. (Q to S) Representative images of JC-1 staining, C11-BODIPY staining, and FerroOrange staining in HUVECs with different treatments. (T) Western blot analysis for ferroptosis biomarkers ACSL4, SLC7A11, and GPX4 in HUVECs with different treatments. *P < 0.05; **P < 0.01; ***P < 0.001. All data are performed in triplicate and at least 3 times.

To eliminate potential off-target effects of pharmacological inhibition, small interfering RNA (siRNA) targeting Piezo1 was constructed to further investigate its role in ferroptosis (Fig. 3D). Based on Western blot analysis, siRNA-Piezo1 #2 was selected for subsequent experiments (Fig. S4A and B). Given the onset time of siRNA action, cells were cotreated with erastin and Doxo for 48 h. Notably, si-Piezo1 treatment improved cellular activity, increased the GSH/GSSG ratio, promoted cell proliferation, and reduced LDH activity (Fig. 3A to C and Fig. S4C and D). TEM imaging revealed that Piezo1 depletion significantly mitigated erastin-induced mitochondrial damage (Fig. 3E). Furthermore, Western blot analysis demonstrated that Piezo1 depletion led to a marked down-regulation of ACSL4 and partially restored the expression of SLC7A11, FTH, and GPX4 (Fig. 3F and Fig. S4G). To assess HUVEC functionality, cell scratch assays, migration experiments, and tube formation assays were performed. Erastin and Doxo treatment inhibited cell migration and tube formation, but these effects were reversed by si-Piezo1 (Fig. 3G to I and Fig. S4I to K). Additionally, Piezo1 knockdown promoted the expression of angiogenesis-related proteins (CD31 and VEGF-A) (Fig. 3F and Fig. S4H). Collectively, these results provide further evidence that Piezo1 deficiency inhibits ferroptosis, restores HUVEC functionality, and supports angiogenesis in erastin- and Doxo-treated HUVECs.

Piezo1 is known to be permeable to Ca^2+^, Na^+^, K^+^, and Mg^2+^ with a preference for Ca^2+^[28]. Fluo-4 AM calcium imaging was employed to assess the effect of Piezo1 channels on erastin- and Doxo-induced responses in HUVECs. Results indicated that erastin induced a significant Ca^2+^ influx, with Doxo showing similar effects on calcium ion flow, which were completely abolished by GsMTx4 treatment (Fig. 3J and K). Fluo-4 AM fluorescence further revealed that the Piezo1 agonist (Yoda1) significantly promoted calcium influx, while si-Piezo1 effectively attenuated Doxo-induced calcium influx (Fig. S4E and F). Calcium ions activate several cellular effectors, including calcium/CaMKII, calcium/calmodulin kinase kinases (CaMKKs), and AKT [29]. Our findings revealed that erastin and Doxo treatments significantly increased CaMKII expression, which was reduced by GsMTx4 (Fig. 3L and M). To systematically explore the role of high Ca^2+^-induced ferroptosis, ethylene glycol tetraacetic acid (EGTA), a calcium chelator, was employed to assess the impact of calcium depletion on ferroptosis. As shown in Fig. 3N to P and Fig. S5A and B, EGTA treatment enhanced HUVEC viability, elevated the GSH/GSSG ratio, reduced LDH release, and promoted cell proliferation in erastin-treated HUVECs. Additionally, EGTA alleviated erastin- and Doxo-induced lipid peroxidation and Fe^2+^ overload and restored mitochondrial function in HUVECs (Fig. 3Q to S and Fig. S5D to F). Western blot assays confirmed that EGTA reversed the effects of erastin and Doxo by normalizing ACSL4 levels and restoring SLC7A11 and GPX4 expression (Fig. 3T and Fig. S5C). These results suggest that EGTA mitigates ferroptosis in erastin- and Doxo-treated HUVECs through its calcium chelation properties. In summary, these results indicate that Piezo1 regulates ferroptosis in HUVECs by modulating calcium channel activity.

### EGTA and Piezo1 channel deficiency may significantly enhance wound closure and promote angiogenesis and alleviate ferroptosis in aging mice

**Fig. 4.**
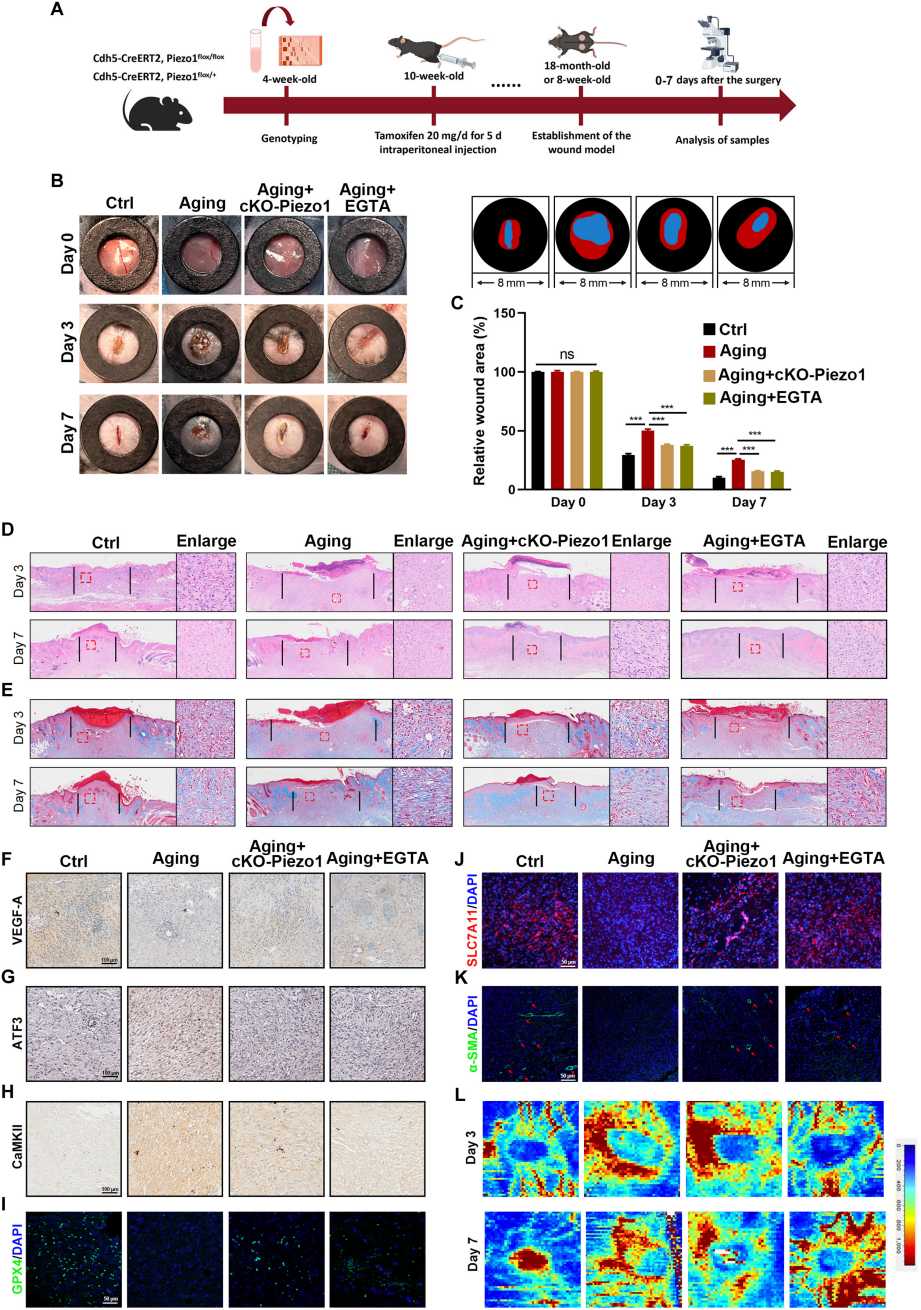
Inhibition of the Piezo1 channel alleviates ferroptosis in HUVECs via a Ca^2+^-dependent mechanism. (A) Schematic of in vivo experiment. (B) Wound images during healing and schematic diagram of wound-healing process. (C) Quantitative data of relative wound area to that of day 0 of the 4 groups. (D and E) H&E and Masson staining images on days 3 and 7. (F to H) Representative IHC images of VEGF-A, ATF3, and CaMKII. (I to K) Representative IF images of GPX4, SLC7A11, and α-SMA. (L) Laser Doppler scanned images of subcutaneous vascular flow and blood supply on wounds at days 3 and 7. ***P < 0.001, ns = no significant. All data are performed in triplicate and at least 3 times.

cKO-Piezo1 mice were generated using a Cre-loxP recombination system, and Piezo1 mRNA knockdown in mouse endothelial cells was confirmed via tail-snip polymerase chain reaction (PCR) (Fig. S6A and B). IF staining revealed minimal Piezo1 expression in cKO-Piezo1 mice (Fig. S6C and D). The experimental workflow is illustrated in Fig. 4A. To assess the role of Piezo1 in wound healing, the mice were divided into 4 groups (*n* = 8): Ctrl (10-week-old mice), Aging (18-month-old mice), Aging + cKO-Piezo1 (18-month-old mice with cKO-Piezo1), and Aging + EGTA. As shown in Fig. 4B and C, cKO-Piezo1 aged mice exhibited faster wound healing on days 3 and 7 compared to the aging group (Fig. 4B and C). Similarly, wound healing in aging mice treated with EGTA was faster on days 3 and 7 than in untreated mice (Fig. 4B and C). Histological evaluation of wound healing and regeneration was performed using H&E and Masson staining on days 3 and 7. H&E staining indicated that the Ctrl group had the shortest wound length (Fig. 4D and E and Fig. S7A). Wound lengths in the Aging + EGTA and Aging + cKO-Piezo1 groups were significantly shorter than in the Aging group (Fig. 4D and E and Fig. S7A). The formation of neovascularization and mature blood vessels was assessed through IHC staining for VEGF-A and IF staining for α-SMA (smooth muscle actin). The number of newly formed and mature blood vessels in the Aging + cKO-Piezo1 and Aging + EGTA groups increased by day 7 compared to the Aging group (Fig. 4F and K and Fig. S7B and G). Blood flow changes around the wound sites during healing were analyzed using the laser Doppler technique, revealing blood flow flux rather than velocity. Both the Aging + cKO-Piezo1 and Aging + EGTA groups exhibited higher blood flow volumes at the wound edge and bed on days 3 and 7 compared to the Aging group (Fig. 4L and Fig. S7H). Furthermore, expression levels of anti-ferroptosis-related proteins (GPX4 and SLC7A11) were elevated in both the Aging + cKO-Piezo1 and Aging + EGTA groups compared to the Aging group (Fig. 4I and J and Fig. S7E and F). Expression of CaMKII was also reduced in the Aging + cKO-Piezo1 and Aging + EGTA groups relative to the Aging group (Fig. 4H and Fig. S7D). Collectively, these results suggest that Piezo1 channel deficiency and EGTA treatment significantly enhance wound closure, promote angiogenesis, and alleviate ferroptosis in aging mice.

### ATF3 involved Piezo1-mediated ferroptosis of senescent HUVECs

**Fig. 5.**
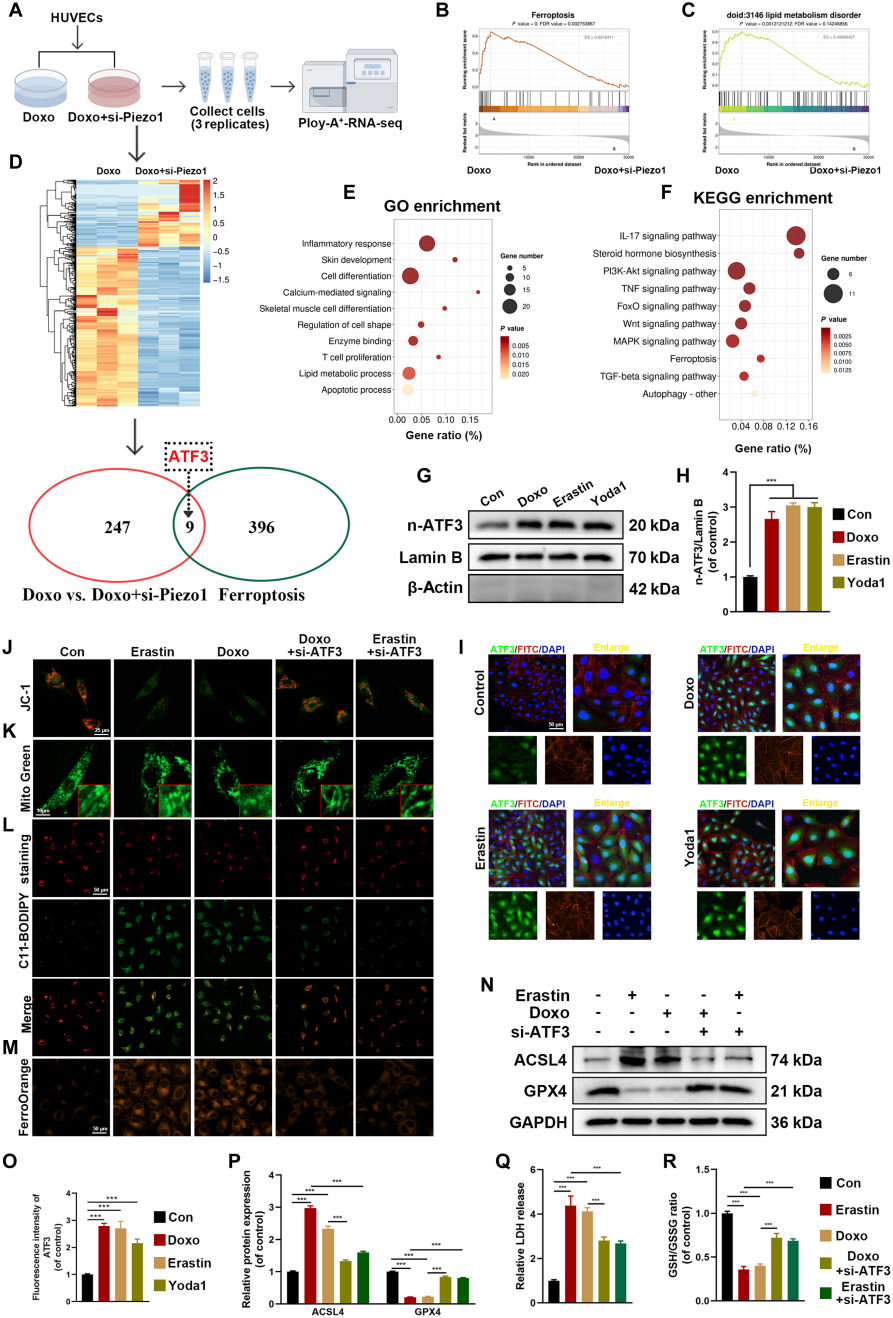
Blockade of ATF3 protects against ferroptosis in erastin- and Doxo-treated HUVECs. (A) Schematic diagram of RNA sequencing design and sample preparation procedures. (B and C) GSEA shows ferroptosis and lipid metabolism in Doxo group and Doxo + si-Piezo1 group. (D) Venn diagram showing these overlapping genes between 2 clusters (Doxo versus Doxo + si-Piezo1 and Ferroptosis) to obtain the intersection of the 2 DEGs. (E and F) GO and KEGG enrichment analyses showing calcium-mediated signaling, lipid metabolic process, and ferroptosis associated with these DEGs. (G and H) Western blot analysis for n-ATF3 in HUVECs with different treatments. (I and O) Representative IF images and quantification analysis of ATF3. (J to M) Representative images of JC-1 staining, Mito Green, C11-BODIPY staining, and FerroOrange staining in HUVECs with different treatments. (N and P) Western blot analysis for ferroptosis biomarkers ACSL4 and GPX4 in HUVECs with different treatments. (Q) LDH release of HUVECs with different treatments for 24 h. (R) GSH/GSSG ratio was measured in HUVECs with different treatments. ***P < 0.001. All data are performed in triplicate and at least 3 times.

To further elucidate the specific mechanism by which Piezo1 regulates ferroptosis in senescent HUVECs, RNA sequencing was performed. The samples were categorized into 2 groups: Doxo and Doxo + si-Piezo1 (Fig. 5A). DEGs were analyzed to assess up-regulation and down-regulation following si-Piezo1 treatment. A total of 247 DEGs were identified between the 2 groups (logFC > 1.5 or logFC < −1.5) (Fig. 5D). GSEA revealed a significant reduction in the expression of genes related to ferroptosis and lipid metabolism in the Doxo + si-Piezo1 group, suggesting that Piezo1 plays a critical role in Doxo-induced ferroptosis (Fig. 5B and C). Further enrichment analysis via GO and Kyoto Encyclopedia of Genes and Genomes (KEGG) highlighted calcium-mediated signaling, lipid metabolism, and ferroptosis pathways as significantly enriched among the DEGs (Fig. 5E and F). To explore ferroptosis-related genes, the FerDB database (http://www.zhounan.org/ferrdb) was used to identify ferroptosis-related DEGs. The intersection of ferroptosis-related DEGs and the 247 identified DEGs yielded 9 genes, including ATF3, Aloxe3, Akr1c1, and others (Fig. 5D). ATF3, a transcription factor, was the only one among the 9 genes. Numerous studies have demonstrated the critical role of transcription factors in regulating lipid peroxidation and ferroptosis, and recent research highlights the involvement of the ATF3/SLC7A11 pathway in ferroptosis [30,31]. RNA sequencing results indicated that Doxo-induced ATF3 pathways were regulated by siRNA-Piezo1, suggesting that ATF3 might be implicated in Piezo1-mediated ferroptosis in HUVECs. We utilized siRNA to knock out the expression of ATF3 (Fig. S8A and B). Western blot and IF analyses revealed that Doxo, erastin, and Yoda1 (a specific Piezo1 agonist) treatment increased n-ATF3 levels in HUVECs (Fig. 5G to I and O). To validate the role of ATF3 in ferroptosis, JC-1 staining, C11-BODIPY staining, and FerroOrange staining were conducted, along with siRNA-ATF3 treatment. The results showed that ATF3 knockdown restored MMP and morphology, and reduced intracellular lipid peroxidation and iron accumulation induced by Doxo and erastin (Fig. 5J to M and Fig. S8C to E). Western blot analysis further revealed that ATF3 knockdown decreased ACSL4 expression, recovered the expression of GPX4, reduced LDH release, and improved GSH/GSSG ratio (Fig. 5N and P to R). In terms of cellular function, ATF3 knockdown restored HUVEC functionality by enhancing cell migration and promoting tube formation (Fig. S8F to K). These results collectively suggest that the ATF3 pathway plays a role in Piezo1-mediated ferroptosis in Doxo- and erastin-treated HUVECs.

### Elevated intracellular Ca^2+^ levels stimulate ferroptosis through the activation of the CaMKII/ATF3 signaling pathway

**Fig. 6.**
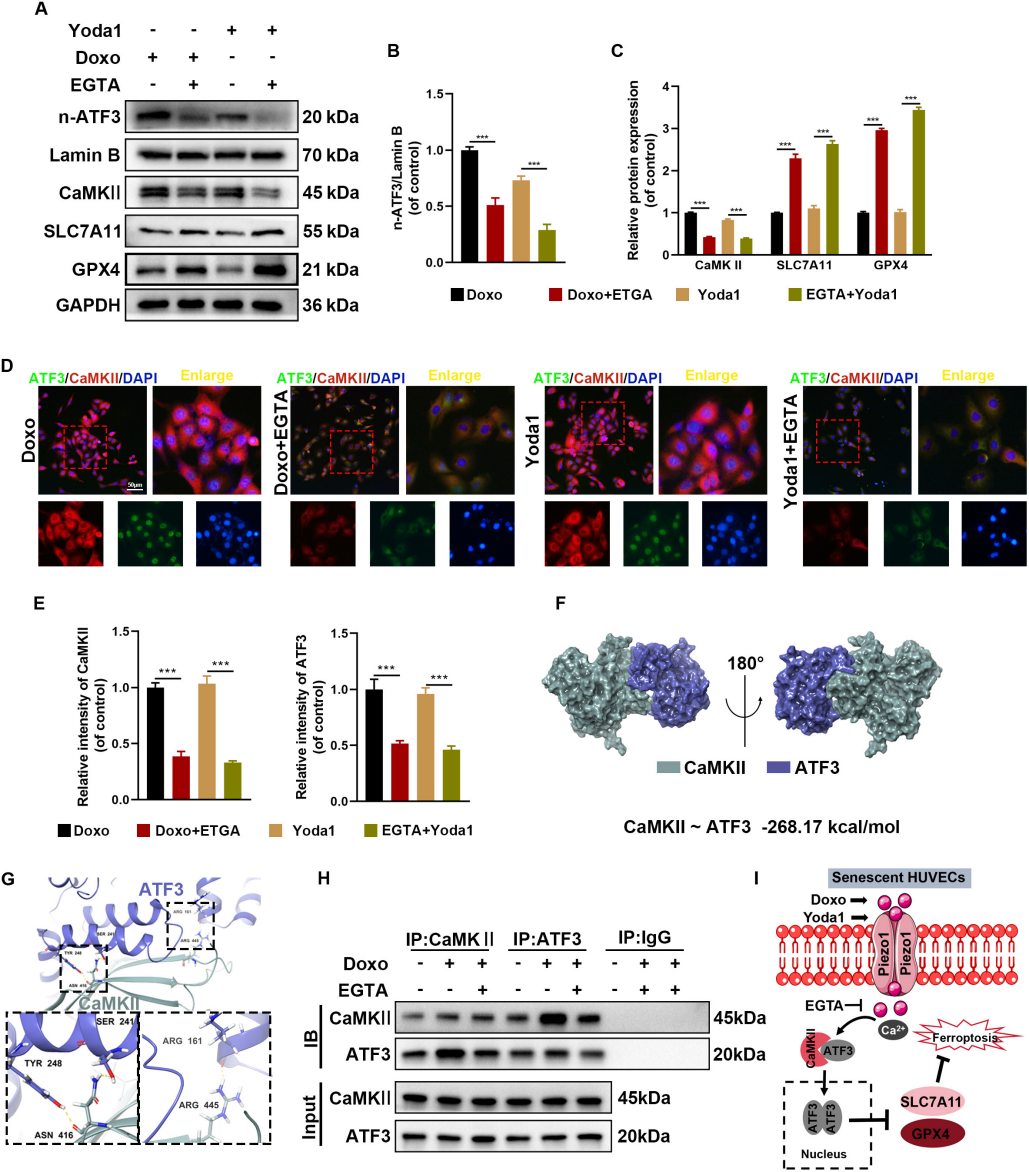
Activation of Piezo1 by Yoda1 facilitates CaMKII-ATF3 interaction in HUVECs. (A to C) Western blot analysis for n-ATF3, CaMKII, SLC7A11, and GPX4 in HUVECs. (D and E) Representative IF images and quantification analysis of CaMKII and ATF3. (F) Three-dimensional binding structure of CaMKII and ATF3 determined via molecular modeling and docking studies. (G) Close-up view of hydrogen bonding between CaMKII and ATF3. (H) The interaction between CaMKII and ATF3 in HUVECs was analyzed by Co-IP. (I) Schematic diagram of the effects of Doxo and EGTA on the binding between CaMKII and ATF3. ***P < 0.001. All data are performed in triplicate and at least 3 times.

The impact of elevated intracellular Ca^2+^ levels on CaMKII and ATF3 was further validated by exposing HUVECs to Yoda1, a well-established agent for increasing intracellular Ca^2+^ concentrations [32]. EGTA treatment inhibited the nuclear translocation of ATF3 in both Doxo- and Yoda1-treated HUVECs, simultaneously restoring the expression of SLC7A11 and GPX4 (Fig. 6A to C). IF analysis of ATF3 and CaMKII corroborated the Western blot results (Fig. 6D and E). Molecular dynamics simulations of the CaMKII-ATF3 heterodimer were performed to explore the interaction interface between the 2 proteins. The simulations revealed 2 binding sites with a binding energy of −268.17 kcal/mol (Fig. 6F). Specifically, Tyr^248^ and Ser^241^ of ATF3 interact with Asn^416^ of CaMKII, while Arg^161^ of ATF3 forms hydrogen bonds with Arg^445^ of CaMKII (Fig. 6G). Notably, CaMKII and ATF3 exhibited interactions under various treatment conditions in HUVECs (Fig. 6H). These results suggest that Piezo1 potentially regulates the interaction between CaMKII and ATF3 by modulating Ca^2+^ levels, thereby influencing ferroptosis in senescent HUVECs (Fig. 6I).

### The overexpression of ATF3 promotes ferroptosis through the repression of the Xc-regulatory system

**Fig. 7.**
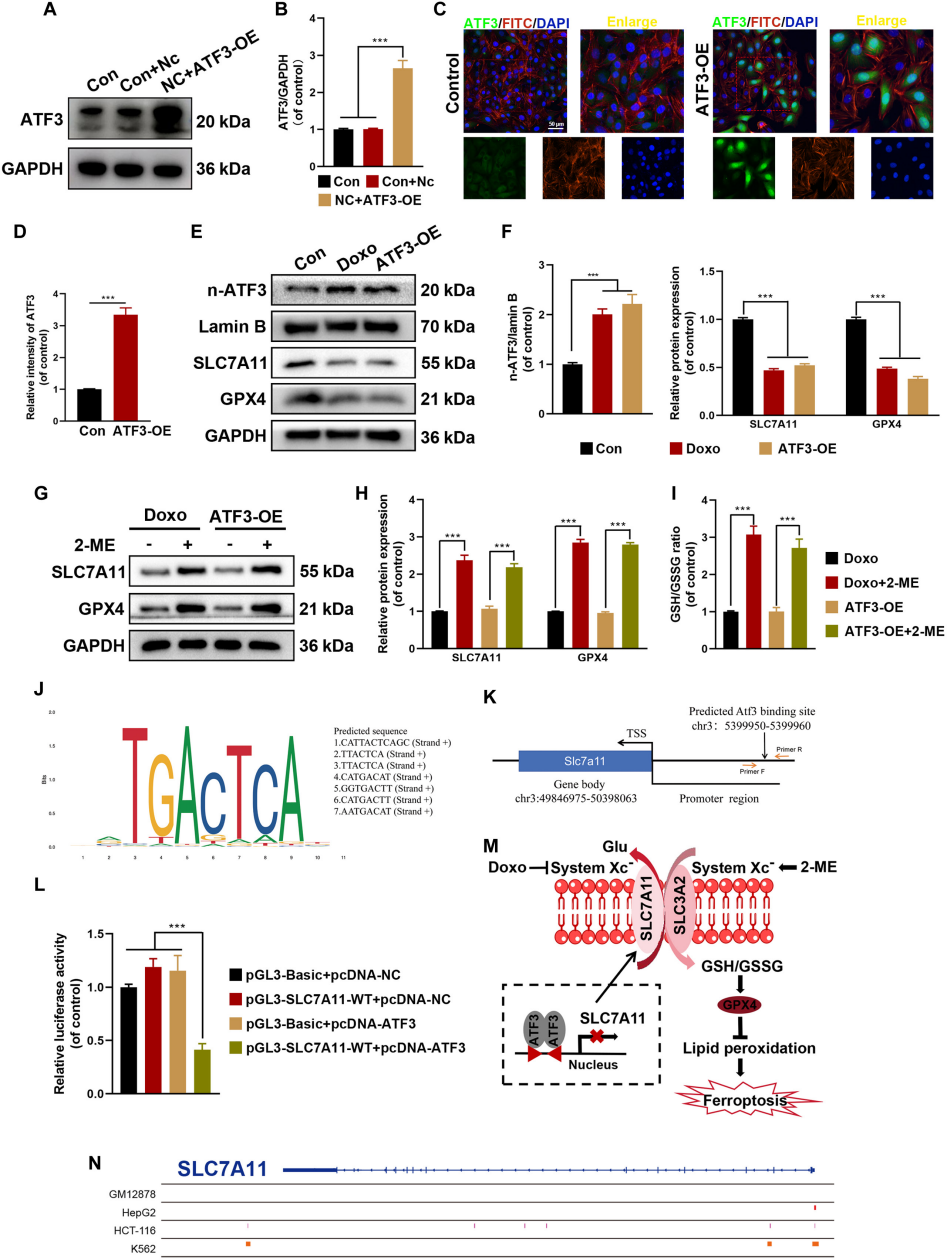
ATF3 represses SLC7A11 expression by regulating system Xc^−^. (A and B) Western blot analysis for ATF3-OE in HUVECs. (C and D) Representative IF images and quantification analysis of ATF3. (E and F) Western blot analysis for n-ATF3 and ferroptosis biomarkers SLC7A11 and GPX4 in HUVECs with different treatments. (G and H) Western blot analysis for ferroptosis biomarkers SLC7A11 and GPX4 in HUVECs with different treatments. (I) GSH/GSSG ratio was measured in HUVECs with different treatments. (J) Predicted ATF3-binding sites in the promoter region of SLC7A11. (K) A schematic illustration depicts the ATF3 motif within the promoter region of the SLC7A11 locus. (L) Luciferase assays were performed in 293T cell. (M) Schematic presentation of a model whereby ATF3 represses SLC7A11 expression to suppress system Xc^−^, and thereby predisposes cells to a state prone to ferroptosis. (N) The chromatin immunoprecipitation sequencing data previously reported were reanalyzed (GSM1917770, ENCSR632DCH_2, GSM803508, GSM803503). ***P < 0.001. All data are performed in triplicate and at least 3 times.

ATF3 was overexpressed (OE) in HUVECs to further investigate its regulatory role in ferroptosis (Fig. 7A to D). Western blot analyses demonstrated that both Doxo treatment and ATF3 overexpression promoted the nuclear translocation of ATF3 and suppressed the expression of SLC7A11 and GPX4 (Fig. 7E and F). Ferroptosis is initiated by system Xc^−^, a heterodimer consisting of SLC7A11 and SLC3A2 [33,34]. 2-Mercaptoethanol (2-ME) converts cystine to cysteine extracellularly, bypassing the need for system Xc^−^ and enhancing cysteine availability for GSH production, thereby increasing the GSH/GSSG ratio [30]. Western blot results indicated that 2-ME restored the expression of anti-ferroptosis proteins SLC7A11 and GPX4 and GSH/GSSH (glutathione disulfide) ratio in HUVECs treated with Doxo or ATF3-OE (Fig. 7G to I). Further analysis of the ATF3 data from the JASPAR database revealed a consensus sequence, “CATT ... CAGC”, within the SLC7A11 promoter region, likely representing the ATF3-binding site (Fig. 7J and K). Thus, ATF3 regulates the transcription of SLC7A11 by binding with high affinity to its promoter, as indicated by data from GSM1917770, ENCSR632DCH_2, GSM803508, and GSM803503 (Fig. 7N). This interaction was further confirmed through a dual-luciferase reporter assay, which demonstrated that ATF3 significantly down-regulated SLC7A11 gene expression (Fig. 7L). Collectively, these results suggest that ATF3 promotes ferroptosis by modulating the activity of system Xc^−^-dependent transcription factors (Fig. 7M).

### The deficiency of ATF3 promotes wound healing and angiogenesis in aging mice

**Fig. 8.**
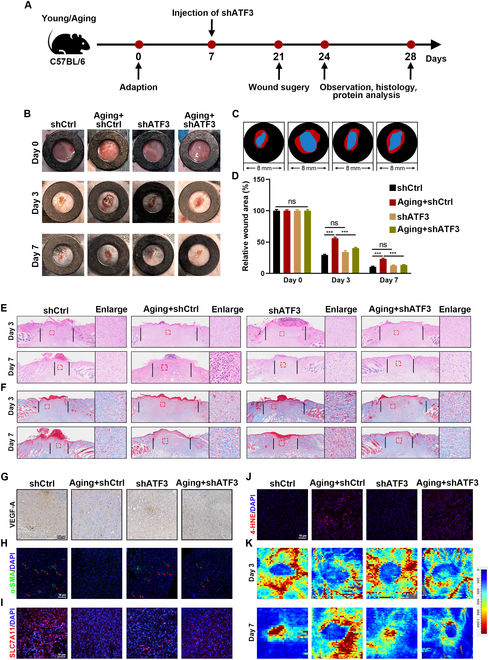
The down-expression of ATF3 inhibits ferroptosis through the regulation of the Xc^−^ system. (A) Schematic of in vivo experiment. (B and C) Wound images during healing and schematic diagram of wound-healing process. (D) Quantitative data of relative wound area to that of day 0 of the 4 groups. (E and F) H&E and Masson staining images on days 3 and 7. (G) Representative IHC images of VEGF-A. (H to J) Representative IF images of SLC7A11, α-SMA, and 4-HNE. (K) Laser Doppler scanned images of subcutaneous vascular flow and blood supply on wounds at day 3 and 7. ***P < 0.001. All data are performed in triplicate and at least 3 times.

ATF3 knockdown was achieved by injecting dorsal skin with AAV9 expressing ATF3-specific shRNA to investigate its role in wound-healing progression in mice (Fig. 8A). The mice were divided into 4 groups (*n* = 8): shCtrl (10-week-old mice), Aging + shCtrl (18-month-old mice), shATF3 (10-week-old mice with shATF3), and Aging + shATF3 (18-month-old mice with shATF3). The wound-healing rate was notably faster in the Aging + shATF3 group compared to the Aging + shCtrl group on both days 3 and 7. The healing rates between the shATF3 and shCtrl groups were comparable (Fig. 8B to D). Histological evaluation using H&E and Masson staining on days 3 and 7 revealed similar trends (Fig. 8E and F and Fig. S9A). Additionally, IF and IHC experiments were performed to assess the role of ATF3 in vivo. The expression of ATF3 was found to be lower in the Aging + cKO-Piezo1 and Aging + EGTA groups compared to the Aging group (Fig. 4G and Fig. S7C). Furthermore, the expression of angiogenesis-related protein (VEGF-A and α-SMA) and anti-ferroptosis-related protein SLC7A11 was more pronounced in the Aging + shATF3 group than in the Aging + shCtrl group, while these proteins were expressed at lower levels in the Aging + shCtrl group (Fig. 8G to I and Fig. S9B to D). Additionally, the Aging + shATF3 group significantly reduced 4-HNE expression than did the Aging +shCtrl group in vivo (Fig. 8J and Fig. S9E). Blood flow and newly formed vascular networks at the wound site were also evaluated using laser Doppler blood flowmetry to visualize the reconstruction of the microvascular network. Increased redness indicated higher blood flow, and intriguingly, blood flow intensity was significantly greater in the Aging + shATF3 group compared to the Aging + shCtrl group (Fig. 8K and Fig. S9F). These results collectively suggest that ATF3 deficiency promotes wound healing and angiogenesis in aging mice.

## Discussion

Cell function in elderly patients’ wounds is compromised by factors such as decreased skin elastic fibers, reduced collagen fibers, thinning of the epidermis and dermis, and impaired microcirculation [35,36]. Researchers are increasingly focusing on elucidating the molecular mechanisms that could accelerate wound healing in the elderly. The senescence-associated secretory phenotype (SASP) plays a key role in regulating the wound-healing microenvironment in senescent cells, particularly in senescent HUVECs, by influencing cell proliferation, matrix remodeling, and angiogenesis [36]. Ferroptosis, a form of programmed cell death, is a critical factor in the progression of diseases, including impaired wound healing in aging and various other physiological processes [37,38]. However, the exact role and mechanisms of ferroptosis in senescent HUVECs remain poorly understood. This study is the first to demonstrate significant ferroptosis in senescent HUVECs and aging wounds, alongside elevated levels of Piezo1 in senescent HUVECs, wounds of elderly patients, and aging mice. Furthermore, inhibiting ferroptosis in senescent HUVECs was shown to promote wound healing in aging mice. Our findings suggest that Piezo1 modulates ferroptosis during wound healing in the elderly, making it a potential therapeutic target for aging populations. Mechanistically, both genetic and pharmacological inhibition of Piezo1 reduced ferroptosis and promoted wound healing in senescent HUVECs through the CaMKII/ATF3/SLC7A11 signaling pathway, highlighting the promise of Piezo1-mediated ferroptosis as a therapeutic approach for wound healing in the elderly.

Several factors, including inflammation, immune cell polarization, and oxidative stress, influence wound healing in the elderly, also affecting various cell types critical to the healing process, such as fibroblasts, endothelial cells, and keratinocytes [6]. Ferroptosis, characterized by elevated ROS levels and lipid peroxidation in wound tissues of elderly patients, is increasingly recognized as a widespread phenomenon in aging [39,40]. Previous studies have shown that administration of Fer-1 alleviates ferroptosis in HUVECs exposed to high glucose, accelerating diabetic wound healing. Moreover, subcutaneous administration of Fer-1 or the iron chelator deferoxamine has been shown to reduce ferroptosis and promote wound healing in HUVECs [9,14]. Several studies also indicate that Doxo induces ferroptosis and senescence both in vitro and in vivo [26,41,42]. Senescent HUVECs undergoing ferroptosis during wound healing necessitate cell-specific targeted therapies to minimize adverse effects. Our findings demonstrate that ferroptosis is significantly prevalent in senescent HUVECs from elderly patients and aging mice compared to younger controls. Therefore, inhibiting ferroptosis in senescent HUVECs enhances wound healing in aging mice.

Piezo1, a cation channel membrane protein, plays a pivotal role in regulating ion influx, signal transduction, and several pathophysiological processes, including cellular proliferation, apoptosis, and inflammation. Oxidative stress is known to promote Ca^2+^ influx through Piezo1, contributing to the development of various diseases associated with pathological conditions. Activation of Piezo1 has been shown to induce chondrocyte death by mediating Ca^2+^ overload and lipid ROS production, whereas inhibition of Piezo1 alleviates oxidative damage [43]. Several calcium channel blockers, including amlodipine, verapamil, diltiazem, nifedipine, and azelnidipine, have been reported to enhance acute wound healing by increasing blood flow to the wound area and stimulating growth factor production [44,45]. Furthermore, Xiang et al. [46] established a correlation between Piezo1, iron overload, and ferroptosis. Previous studies have demonstrated that blocking Piezo1 accelerates wound healing in elderly individuals [47]. In line with these findings, our data suggest that inhibiting Piezo1 significantly suppresses ferroptosis in HUVECs induced by erastin or Doxo and promotes wound healing in senescent cells. Additionally, the inhibition of Piezo1-mediated Ca^2+^ influx effectively alleviates ferroptosis, highlighting that Piezo1 channels regulate ferroptosis in senescent HUVECs.

Mitochondria are essential for regulating cellular metabolism, including adenosine triphosphate (ATP) production, ROS generation, calcium balance, and apoptosis. They also play a critical role in redox homeostasis, cellular metabolism, and iron regulation during ferroptosis [48]. Jang et al. [49] demonstrated that the activation of ferroptosis impairs mitochondrial bioenergetics and accelerates the degradation of GSH, while Liang et al. [50] reported that mitochondrial ROS (mtROS) further activates ferroptosis in renal cells. Growing evidence indicates that changes in MMP and mtROS are central to mitochondrial involvement in ferroptosis. Consequently, therapies targeting ferroptosis may benefit from strategies aimed at restoring mitochondrial function. Our experimental results support this notion, showing that both pharmacological inhibition and genetic knockout of Piezo1 can rescue mitochondrial morphology, restore MMP in senescent endothelial cells, and reduce mtROS.

The cystine-glutamate transporter SLC7A11 is critical for intracellular GSH synthesis during ferroptosis, helping to mitigate ROS formation [51,52]. Transcription factors are widely believed to regulate this process. While previous research has elucidated the role of Piezo1 in ferroptosis regulation, specific studies investigating the downstream transcriptional regulation of Piezo1 during ferroptosis remain scarce [15,46]. The ATF3 gene, a member of the ATF/CREB family, is rapidly induced by various cellular stresses, including DNA damage, oxidative stress, and injury [53]. Several studies indicate that ATF3 acts as a transcriptional activator or repressor, modulating intracellular ROS levels [30,54]. While ATF3 has traditionally been studied for its role in endoplasmic reticulum stress, its involvement in ferroptosis remains unclear. Notably, ATF3 suppresses the activity of SLC7A11. Accumulating evidence has shown that ATF3 contributes to ferroptosis induced by erastin by inhibiting SLC7A11 transcription [55]. In this study, inhibiting ATF3 prevented lipid peroxidation and alleviated ferroptosis in senescent HUVECs. ATF3 promotes ferroptosis by regulating system Xc^−^ expression, as evidenced by blocking ATF3 with 2-ME. Through data analysis, the ATF3-binding site was identified within the SLC7A11 promoter. To confirm that ATF3 regulates SLC7A11 transcription, promoter analysis, Western blot, IF staining, and dual-luciferase reporter assays were performed. However, further investigation is needed to validate these findings. The activation of Piezo1 (via Yoda1) increased ATF3 expression and reduced SLC7A11 expression, suggesting that the CaMKII/ATF3/SLC7A11 signaling pathway may regulate ferroptosis in senescent HUVECs. Although our experimental data indicate that inhibiting Piezo1 plays a positive role in promoting wound healing in aging, several limitations remain in this study. First, larger human sample studies are necessary for verification. Second, translating basic research into clinical applications is complex and lengthy. In a clinical context, small molecule inhibitors or gene editing techniques (e.g., CRISPR-Cas9) could precisely regulate Piezo1 expression or function. Additionally, nanodelivery systems (e.g., liposomes or polymer nanoparticles) could be used to target and deliver inhibitors directly to wound sites, enhancing local drug concentrations while minimizing potential systemic effects. However, several challenges remain before clinical applications can be realized, including potential side effects, and further validation of Piezo1 inhibitor safety and efficacy in humans is required.

## Conclusion

**Fig. 9.**
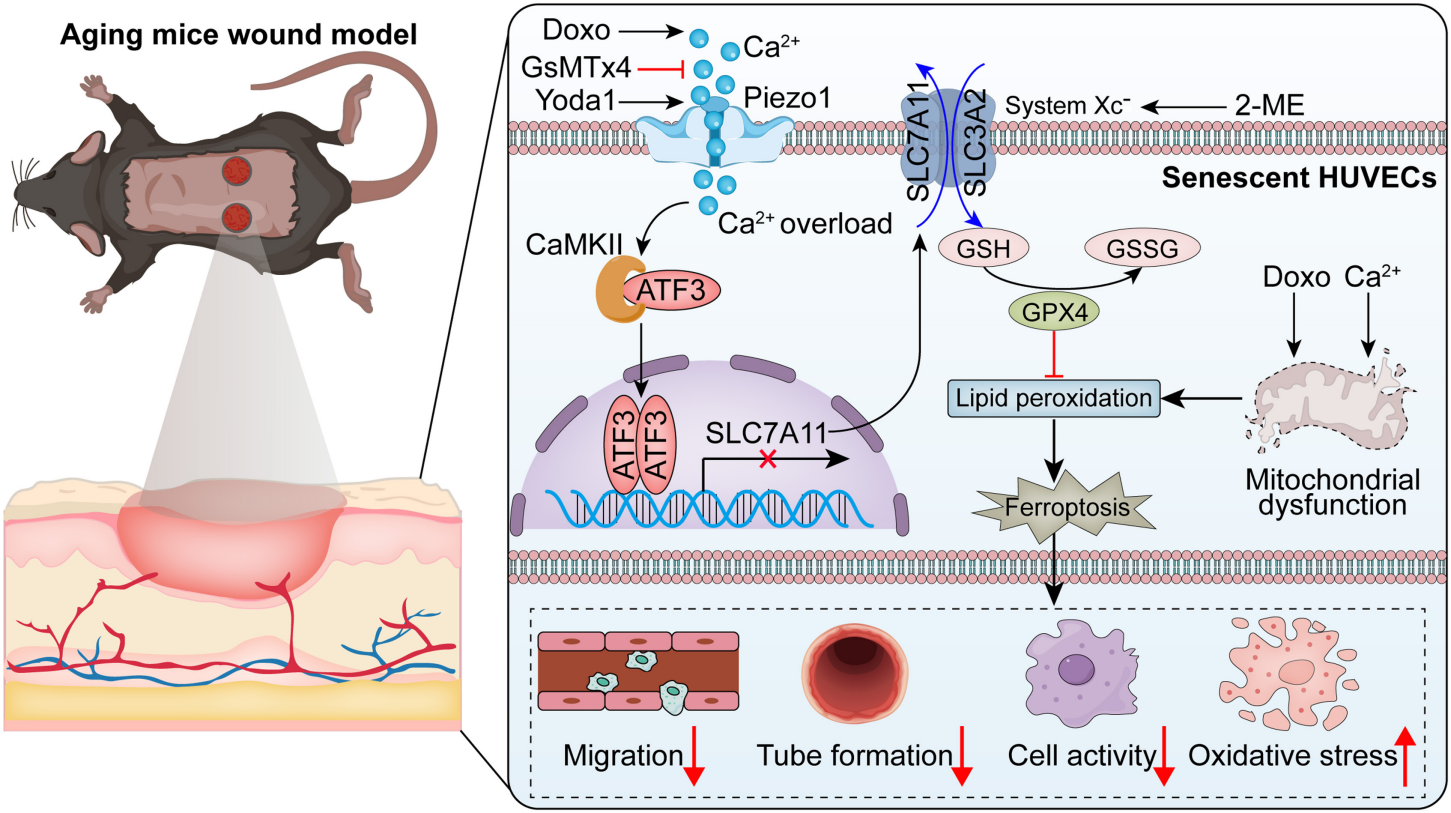
The deficiency of ATF3 promotes wound healing and angiogenesis in aging mice. Piezo1 up-regulated by calcium ion influx triggers ATF3 nuclear translocation via activation of CaMKII, thus aggravating the accumulation of ROS and lipid peroxidation, and eventually leading to ferroptosis in senescent HUVECs.

In summary, this study provides the first evidence that Piezo1 modulates ferroptosis in senescent HUVECs and influences wound-healing processes. GsMTx4 and EGTA were identified as potential ferroptosis inhibitors. Additionally, Piezo1 absence promoted wound healing in senescent HUVECs by alleviating ferroptosis. Thus, targeting Piezo1 may serve as an effective therapeutic strategy to accelerate wound healing in the elderly (Fig. 9).

## Materials and Methods

### Human specimen acquisition

Young and elderly patients (*n* = 4) undergoing surgery for various reasons, including accidental trauma, were classified into the young and aging groups. Adequate wound and peri-wound tissue samples were collected from these patients for analysis. The research protocol was approved by the Ethics Committee of the First Affiliated Hospital of Wenzhou Medical University (KY2023-294).

### Culture of HUVECs

HUVECs were obtained from ScienCell Corporation (Shanghai, China) and cultured in Dulbecco’s modified Eagle’s medium (DMEM; Gibco, Grand Island, NY, USA) supplemented with 10% (v/v) fetal bovine serum and 1% (v/v) penicillin/streptomycin. The cultures were maintained at 37 °C in a humidified incubator with 5% CO_2_.

### Cell treatment

To induce senescence, HUVECs were treated with Doxo (100 nM, MedChemExpress, HY-15142A, China) [26]. The culture medium was refreshed every 2 d during the Doxo treatment. In vitro experiments were conducted by treating HUVECs with erastin (1 μM, MedChemExpress, HY-15763, China), GsMTx4 (2.5 μM, HY-P1410, China), EGTA (20 mM, HY-D0861, China), Yoda1 (5 μM, HY-18723, China) and 2-ME (20 μM, HY-Y0326, China). All reagents were of analytical grade and met all cell culture standards.

### SA-β-Gal staining

HUVECs were fixed with 0.2% formaldehyde (4 °C) for 15 min at room temperature (RT). The cells were washed 3 times with phosphate-buffered saline (PBS) and incubated with SA-β-Gal staining solution (37 °C, pH 6.0) for 12 h. SA-β-Gal-positive cells were observed and quantified using an inverted phase contrast microscope (Leica, Germany).

### Cell proliferation and LDH assay

For cell viability assays, HUVECs were seeded at a density of 5 × 10^3^ cells/well in 96-well culture plates. The cells were treated with CCK-8 reagent (Beyotime, catalog no. C0037, China) for 2 h at 37 °C in the dark, and the absorbance was measured at 450 nm using a microplate reader. Cell proliferation was further assessed using the EdU Cell Proliferation Kit (Beyotime, catalog no. C0071L, China) according to the supplier’s instructions. The HUVECs were treated with EdU for 4 h, fixed with 4% polyformaldehyde for 30 min, and stained with Hoechst for nuclear staining. The stained cells were then observed and photographed under a microscope (Leica, Germany). For cytotoxicity detection, HUVECs were seeded in 24-well plates at a density of 5 × 10^4^ cells/well and 50 μl of supernatant was transferred to 96-well plates. After incubating with 5% Triton X-100 for 30 min, the absorbance at 492 and 620 nm was measured using the Cytotoxicity Detection Kit (Beyotime, catalog no. C0016, China).

### Analysis of the GSH/GSSH ratio

HUVECs were seeded in a 6-well plate at a density of 2 × 10^5^ cells per well. The GSH and GSSG levels were measured using the GSSG/GSH Quantification Kit (Dojindo, catalog no. G263, China) following the treatment.

### Mitochondrial function assays

mtROS production and membrane potential were assessed using Mito Green (Beyotime, catalog no. C1048, China) and JC-1 (Beyotime, catalog no. C2003S, China) fluorescence probes. Fluorescent images were captured in randomly selected areas (Leica, Germany), with red and green fluorescence indicating healthy mitochondria, reflecting high and low membrane potentials, respectively.

### C11-BODIPY and FerroOrange staining

Lipid peroxidation levels were determined using the C11-BODIPY probe (Invitrogen, catalog no. D3861, USA). HUVECs were incubated for 30 min with a working solution of C11-BODIPY (1 mg/ml) post-treatment. To detect intracellular Fe^2+^, the FerroOrange probe was utilized. HUVECs were washed with PBS and incubated with a FerroOrange working solution (1 μM) for 30 min. Labeled cells were observed using confocal scanning microscopy (Leica, Germany).

### Western blot analysis

Protein extraction was performed using radioimmunoprecipitation assay (RIPA) buffer (Beyotime, catalog no. P0013B, China) supplemented with phosphatase protease inhibitors (Beyotime, catalog no. ST505, China). Protein concentrations were measured using the BCA Protein Assay Kit (BCA) (Beyotime, catalog no. P0010, China) following centrifugation at 12,000*g* for 30 min. Proteins separated by sodium dodecyl sulfate–polyacrylamide gel electrophoresis (SDS-PAGE) were transferred onto PVDF membranes. The membranes were adequately blocked with 10% skim milk solution for 1 h and then incubated overnight with specific primary antibodies (Table S3). Following the primary antibodies were applied at room temperature for 1 h with horseradish peroxidase-conjugated secondary antibodies, goat anti-rabbit HRP IgG (FDR007) and goat anti-mouse HRP IgG (FDM007) were obtained from Hangzhou Fude Biological Technology. The immunoreactive bands were detected with an enhanced chemiluminescence (ECL) substrate (Meilunbio, catalog no. MA0186, China) and visualized with a chemiluminescent substrate (Bio-Rad, USA).

### Immunofluorescence

HUVECs were cultivated at 37 °C, permeabilized with 0.1% Triton X-100 in PBS for 15 min, and blocked with 1% goat serum at RT for 1 h to eliminate nonspecific binding. The cells were then incubated overnight at 4 °C with primary antibodies, followed by 3 PBS washes and a 1-h incubation with fluorescent secondary antibodies. The nuclei were stained with 4′,6-diamidino-2-phenylindole (DAPI) for 5 min at RT. Images were acquired using a fluorescence microscope, and the IF data were quantitatively analyzed using Image-Pro Plus 2D Software (Rockville, MD, USA).

### Calcium imaging

For calcium imaging, HUVECs were pretreated with Doxo or erastin in the presence or absence of GsMTx4 and then loaded with Fluo-4 AM (5 μM; Beyotime, catalog no. S1062, China) for 30 min. Calcium imaging was performed using a Leica fluorescence microscope.

### Transmission electron microscopy

HUVECs were fixed in 2.5% glutaraldehyde, washed in 1% osmium tetroxide, and dehydrated using a series of ethanol and acetone solutions. The samples were embedded in resin and infiltrated with anhydrous acetone and epoxy resin for 48 h. TEM (Thermo, USA) was utilized to examine ultrathin sections prepared using an ultra-microtome (Leica, Germany).

### siRNA transfection

siRNAs targeting Piezo1 and ATF3 were purchased from Invitrogen (Carlsbad, CA, USA). The HUVECs were transfected with siRNA when their confluence reached 30 to 50 %. After 12 h, >95% of the cells remained viable. Western blot analysis was performed to confirm the transfection efficiency.

### Co-immunoprecipitation

A co-immunoprecipitation (Co-IP) assay was performed using the Classic IP/Co-IP Assay Kit (Invitrogen, catalog no. 88804). Cells were fixed and lysed in a gentle lysis buffer, and 90% of the lysate was incubated with ATF3-specific antibodies (from Beyotime, catalog no. P2171, China) and an immunoglobulin G (IgG) control (same supplier) for 24 h. Complexes were then pelleted, washed 3 times with lysis buffer, and denatured before being loaded onto a denaturing SDS-PAGE gel for analysis.

### Plasmid transfection

The ATF3 gene sequences were cloned into the pEX-3 vector (from GenePharma, China), and cells were transfected with pEX-3-ATF3 using Lipofectamine 3000 (Invitrogen, USA). After a 6-h pretreatment, cells were cultured in a fresh medium for 48 h. Overexpression of ATF3 was confirmed via Western blot analysis.

### Luciferase reporter assay

To evaluate the promoter activity of SLC7A11, 293T cells were cotransfected with the SLC7A11 luciferase reporter plasmid, pcDNA-ATF3, and the Renilla luciferase control plasmid. Luciferase activity was measured using the dual-luciferase assay system (Promega Biotech Co. Ltd., catalog no. E1901, China) 48 h post-transfection.

### Animals

Animal experiments were conducted in compliance with institutional guidelines and approved by the Laboratory Animal Centre of Wenzhou Medical University (wydw2024-0322). The Cdh5-CreERT2 mice obtained from Cyagen (USA) were used for genome editing. Piezo1^flox/+^ mice were generated via electroporation of the embryonic stem cell into the CreERT2 transgenic line, and then mice were crossed to produce the double heterozygous (Piezo1^flox/flox^) strain. This process was repeated to ensure homozygosity. Piezo1^flox/flox^ mice were mated with Cdh5-CreERT2 mice to generate the heterozygous (Cdh5-CreERT2 Piezo1^flox/+^) mice, which were further crossed to produce the double heterozygous (Cdh5-CreERT2 Piezo1^flox/flox^) mice (Fig. S4A). Cdh5-CreERT2 Piezo1^+/+^ mice were designated as the wild-type (WT) group. At 10 weeks of age, 10-week-old Cdh5-CreERT2 Piezo1flox/flox mice were intraperitoneally injected with tamoxifen (20 mg/d × 5 d) (MedChemExpress, ST1681, China) to generate the conditional knockout (cKO) mice. Additionally, healthy 10-week-old C57/BL6 mice and 18-month-old C57/BL6 mice were purchased from the Experimental Animal Center of Wenzhou Medical University. All animals were housed under controlled conditions (12-h light/dark cycle) in pathogen-free environments at 24 ± 2 °C and 55 ± 5% humidity.

### Genotyping

Tail clippings were obtained from 4-week-old mice using the One Step Mouse Genotyping Kit (Vazyme, catalog no. PD101-01, China), and DNA was extracted from the mouse tail clippings according to the kit’s instructions. The primers used for amplification, Piezo1^flox^ and Cdh5-CreERT2, are listed in Table S2. An agarose gel was prepared with 1.5 g of agarose, 100 ml of TAE (Tris–acetate–EDTA) buffer, and 6 ml of Gel Red. Electrophoresis of the amplified DNA was performed on the agarose gel, and images were captured using an Amersham Imager 680 (GE, USA). The Cdh5-CreERT2 genotype was confirmed by a 358-base pair (bp) band, with no band detected for the WT allele. The Piezo1flox/flox genotype appeared as a 238-bp band, the WT genotype (Piezo1^+/+^) appeared as a 204-bp band, and the heterozygous genotype (Piezo1^flox/+^) appeared as 2 bands at 238 and 204 bp. The primers used are detailed in Table S2, and the results are illustrated in Fig. S6B.

### Generation of mice AAV9 vector and injection

The adeno-associated virus 9 (AAV9)-ATF3 shRNA vector was produced by Hanbio Biotechnology (Shanghai, China) with a virus titer of 1 × 10^12^ μg/ml. The AAV9-ATF3 shRNA was administered intradermally to mice as outlined in the experimental design.

### Establishment of the full-thickness skin wound model

A full-thickness skin wound model was established as described previously [56]. Mice were anesthetized with intraperitoneal injections of 1% pentobarbital sodium, followed by depilation using a cream-based hair removal solution. After rinsing with warm water, the mice were allowed to rest. Two 8-mm sterile punch biopsy tools were employed to create full-thickness wounds on the dorsal skin of the mice under anesthesia. Post-surgery, the mice were housed in specific pathogen-free conditions with unrestricted access to food and water. The ambient temperature was maintained at 23 to 27 °C.

### Wound-healing assessment and histological staining

Wound closure was evaluated using digital photography on days 3 and 7. The wound closure time and wound area were quantified using ImageJ software. Wound tissues were excised, fixed in paraformaldehyde, embedded in paraffin, and sectioned between days 3 and 7. The sections were deparaffinized, rehydrated, and stained using H&E and Masson staining kits (Beyotime, catalog no. C0105M, China). Histological analysis was performed using a Leica microscope at 40× and 100× magnifications.

### IHC examination

The sectioned tissues were deparaffinized in xylene for 30 min, rehydrated, and incubated with specific primary antibodies overnight at 4 °C against anti-VEGF-A (1:200), anti-ATF3 (1:200), and anti-CaMKII (1:200). The sections were adequately washed and then incubated with secondary antibodies (goat anti-rabbit IgG or goat anti-mouse IgG, Invitrogen, catalog no. A11012, A-11005, China) for 2 h. The staining was revealed by using DAB (Solarbio, catalog no. DA1010-10, China).

### Assessment of blood flow in the wound area

The blood flow in dorsal wounds was evaluated using a laser Doppler imaging system (Moor Instruments, Axminster, UK) on days 3 and 7 after surgery. MoorLDI Review software was used to assess the reconstruction of the microvascular network.

### Statistical analysis

Statistical analysis was performed using GraphPad Prism 9.4.0. Two-tailed unpaired *t* tests were used for pairwise comparisons between 2 groups, while one-way analysis of variance (ANOVA) was employed for comparisons among multiple groups. Statistical significance was defined as *P* < 0.05 for mean ± SEM. The experiment was performed 3 times, with *n* = 3 samples per experiment.

## Data Availability

The data that were utilized to support the findings of this study can be obtained from the author who corresponds to this work.
