# Supplementary material for: Piezo1-Mediated Ferroptosis Delays Wound Healing in Aging Mice by Regulating the Transcriptional Activity of SLC7A11 through Activating Transcription Factor 3
Source: Research (Wash D C). 2025 Jun 3;8:0718. doi: 10.34133/research.0718 (PMC12133029; doi:10.34133/research.0718)
Supplement: Supplementary 1 — Figs. S1 to S9 Tables S1 to S3 [file research.0718.f1.docx]

**Supplementary Information**

This supporting information includes:

Supplementary Figures S1 to S9

Supplementary Table S1 to S3

**
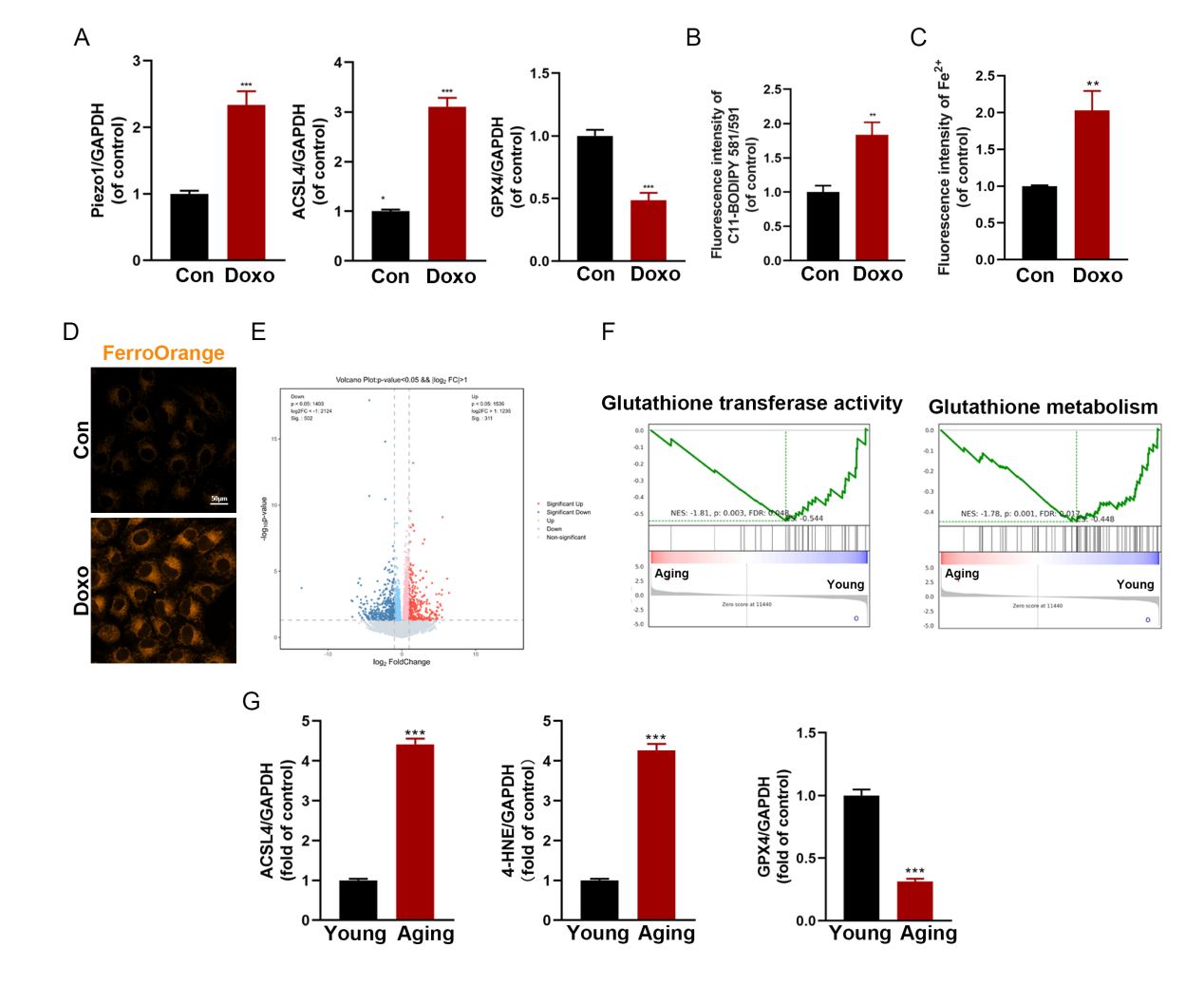
**

**SFig. 1 Doxo-treated HUVECs and aging miouse wounds increased the expression of ferroptosis-related protein and Piezo1.** (A) Quantification analysis of the expression of Piezo1, ACSL4 and GPX4. (B) Quantification analysis of the fluorescence intensity of C11-BODIPY. (C-D) Representative images and quantification analysis of FerroOrange staining. (E) Volcano plot show the differentially expressed genes of the two groups. (F) GSEA showing glutathione transferase activity and glutathione metabolism in young group and aging group. (G) Quantification analysis of the expression of ACSL4, 4-HNE and GPX4. ^*^ indicates a comparison between the two groups. ^**^p < 0.01; ^***^p < 0.001. All data are from n ≥ 3 independent experiments.


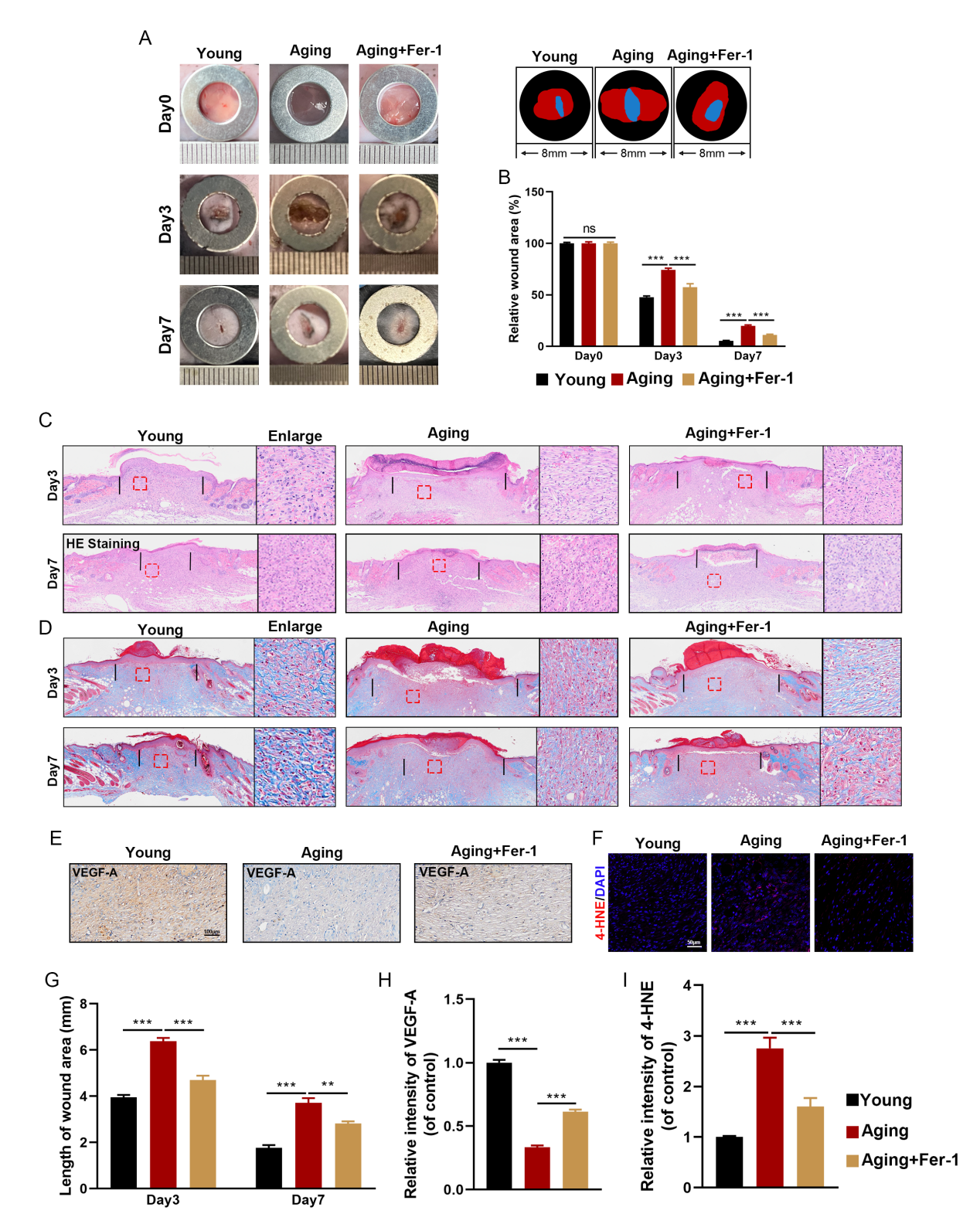


**SFig. 2 The ferroptosis inhibitor Fer-1 promotes wound healing and angiogenesis in aging mice.** (A) Wound images during healing and schematic diagram of wound-healing process. (B) Quantitative data of relative wound area to that of day 0 of the four groups. (C-D and G) H&E and Masson staining images on days 3 and 7, and the quantification analysis of length of wound are in different groups. (E and H) Representative IHC images and quantification analysis of VEGF-A. (F and I) Representative IF images and quantification analysis of 4-HNE. ^*^ indicates a comparison between the two groups. ^***^p < 0.001, ns = no significant. All data are from n ≥ 3 independent experiments.

**
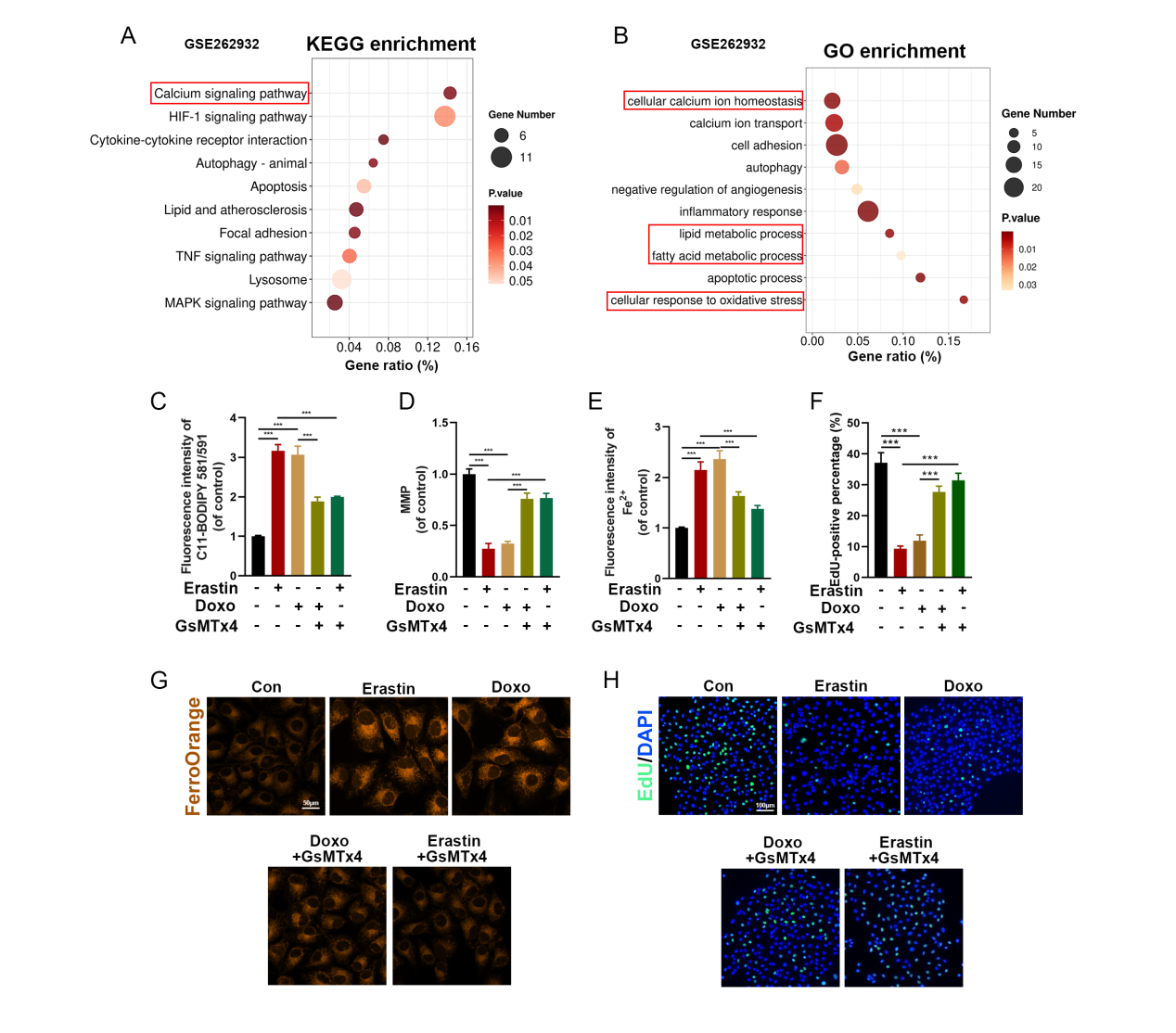
**

**SFig. 3 GsMTx4 inhibits ferroptosis of senescent HUVECs.** (A-B) KEGG and GO analyses of the Genome-wide RNA-Seq dataset (GSE262932). (C) Quantification analysis of the fluorescence intensity of C11-BODIPY. (D) Quantification analysis of the MMP. (E and G) Representative images and quantification analysis of the fluorescence intensity of Fe^2+^. (F and H) Representative images and quantification analysis of the EdU staining. ^*^ indicates a comparison between the two groups. ^***^p < 0.001. All data are from n ≥ 3 independent experiments.


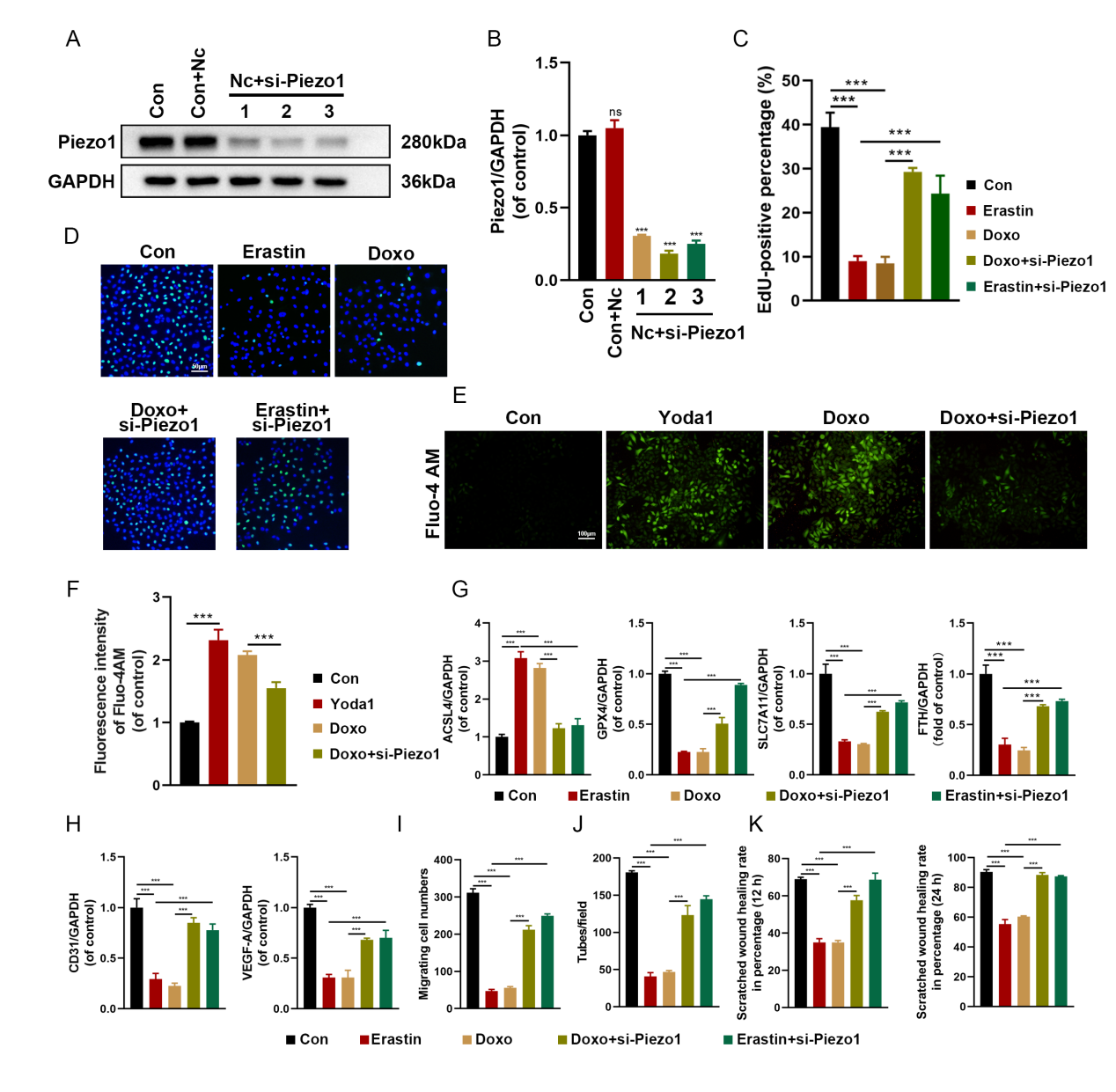


**SFig. 4 Knockdown of Piezo1 inhibited ferroptosis of senescent HUVECs.** (A -B) Western blot analysis for Piezo1 with different treatments. (C-D) Representative images and quantification analysis of the EdU staining. (E-F) Representative images and quantification analysis of Fluo-4 AM staining in HUVECs with different treatments. (G-H) Western blot quantification analysis of ACLS4, GPX4, SLC7A11, FTH, CD31 and VEGF-A in HUVECs with different treatments. (I) Quantification analysis of migrating cell numbers. (J) Quantification of tube-forming ability. (K) Quantification of the scratch migration assay. ^*^ indicates a comparison between the two groups. ^***^p < 0.001, ns = no significant. All data are from n ≥ 3 independent experiments.

**
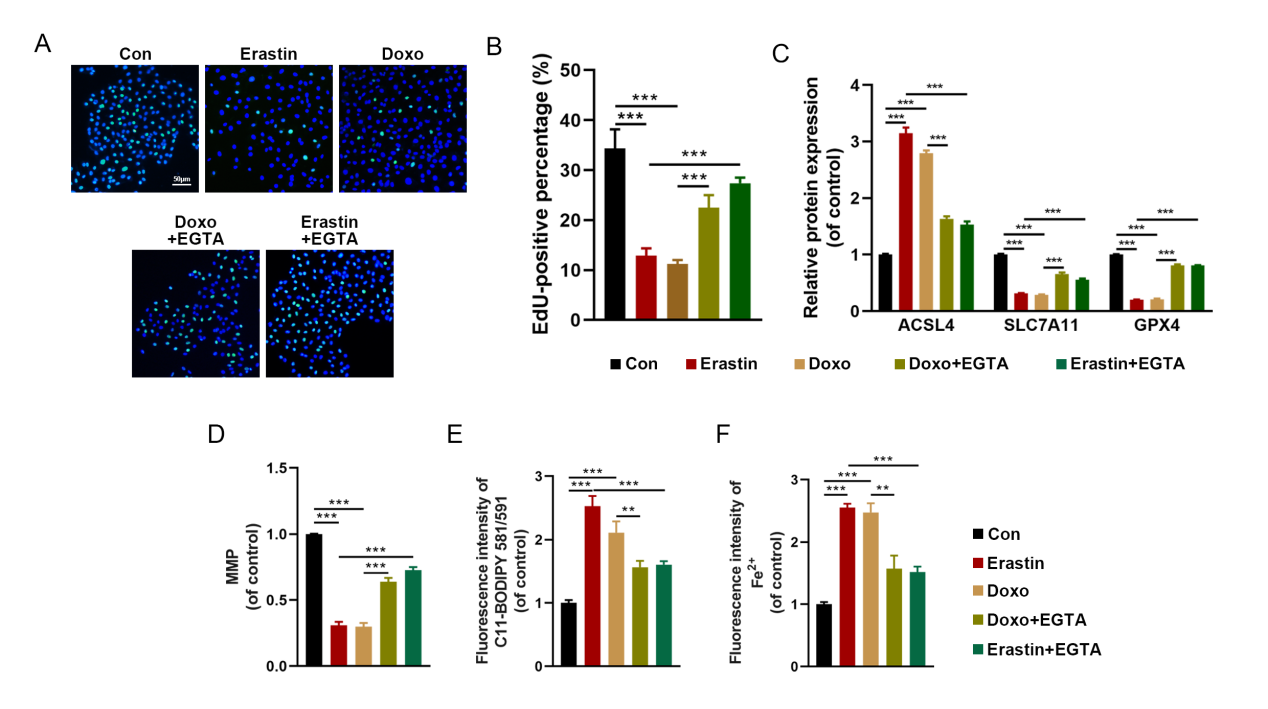
**

**SFig. 5 EGTA inhibits ferroptosis of senescent HUVECs.** (A-B) Representative images and quantification analysis of the EdU staining. (C) Western blot quantification analysis for ferroptosis biomarkers ACSL4, SLC7A11 and GPX4. (D) Quantification analysis of the MMP. (E) Quantification analysis of the fluorescence intensity of C11-BODIPY. (F) Quantification analysis of the fluorescence intensity of Fe^2+^. ^*^ indicates a comparison between the two groups. ^***^p < 0.001, ns = no significant. All data are from n ≥ 3 independent experiments.

**
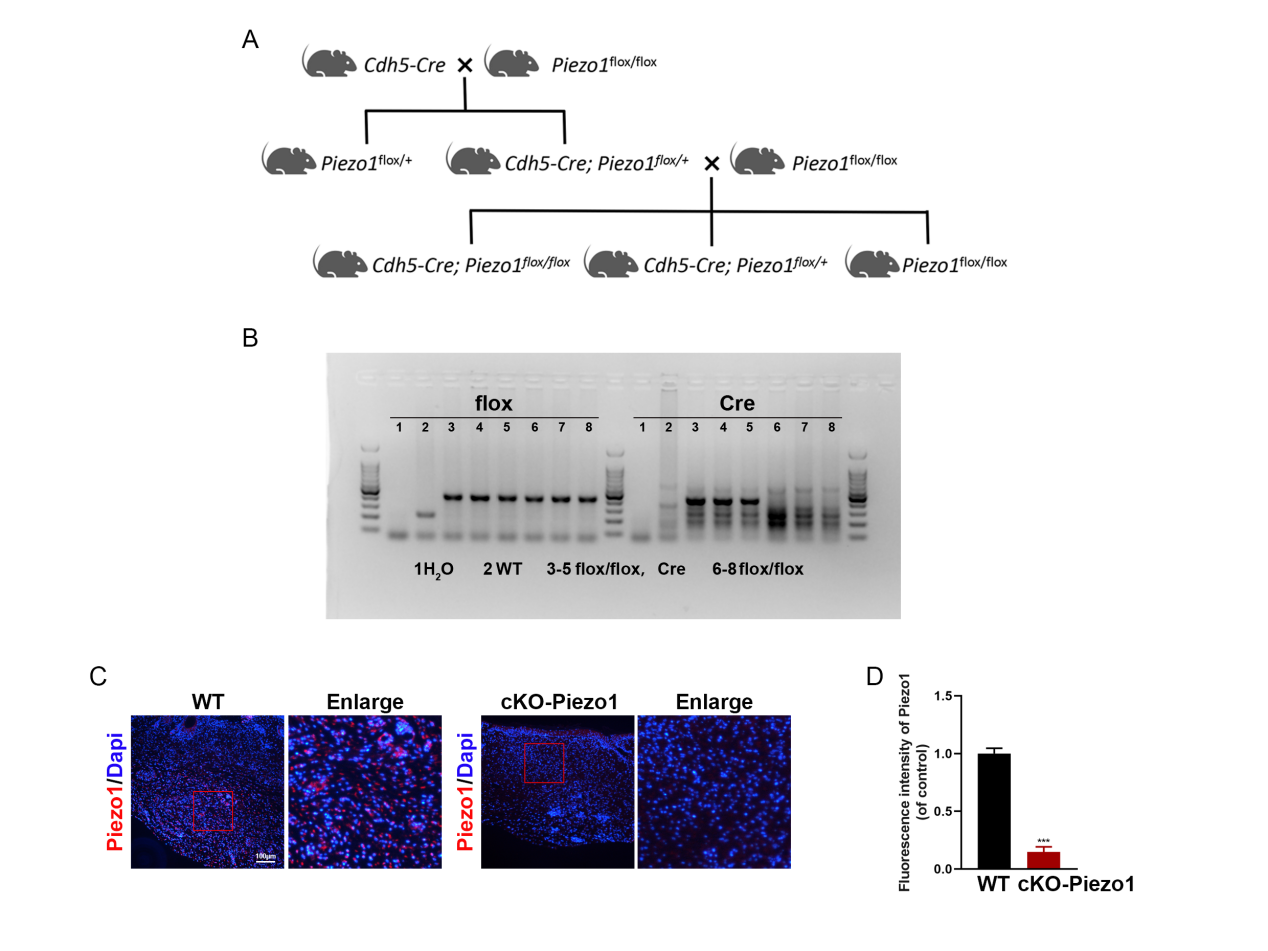
**

**SFig. 6 Breeding strategies and gene identification of mice.** (A) Breeding strategies for cKO-Piezo1 (Piezo1*^flox/flox^* × Cdh5-Cre). (B) Gene identification results. In Cdh5-Cre group, Lane1: H_2_o; Lane2: Wild type; Lane3-5: Piezo1*^flox/flox^*; Lane6-8: Piezo1*^flox/flox.^*. (C-D) Representative images and quantification analysis of the fluorescence intensity of Piezo1 in aging WT and cKO-Piezo1 mice. ^*^ indicates a comparison between the two groups. ^***^p < 0.001, ns = no significant. All data are from n ≥ 3 independent experiments.

**
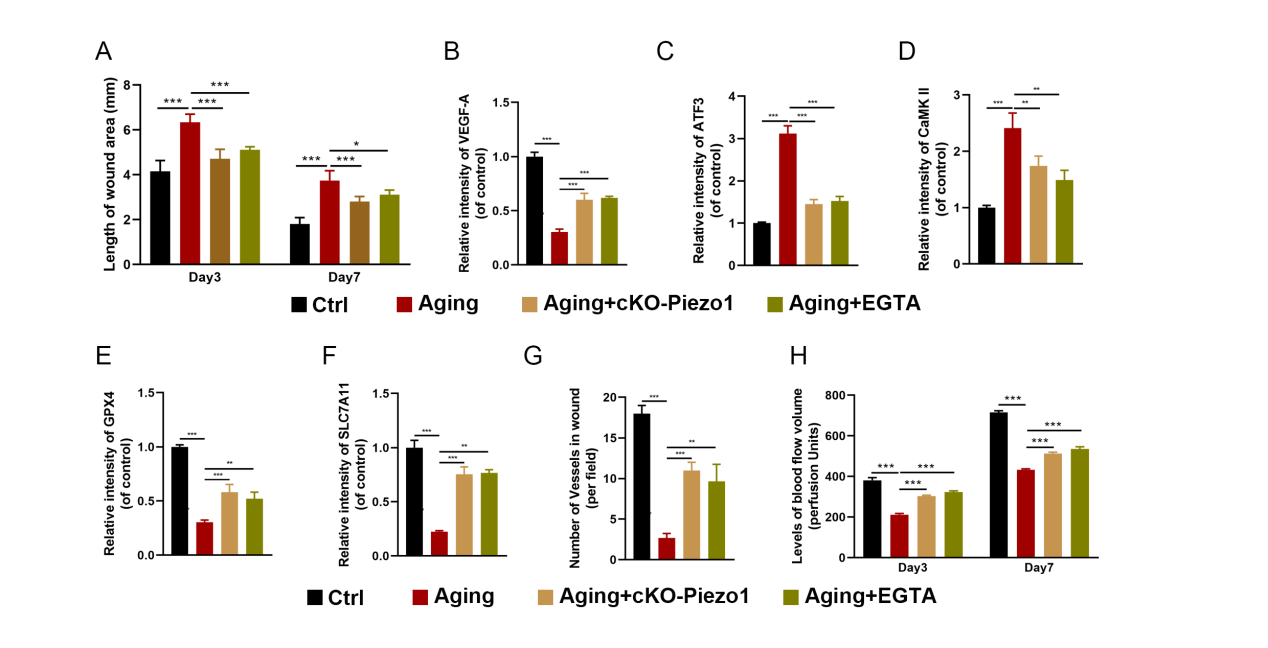
**

**SFig. 7 Knockdown of Piezo1 and EGTA treatment promote aging wound healing and angiogenic.** (A) Quantification analysis of length of wound are in different groups. (B-D) IHC quantification analysis of VEGF-A, ATF3 and CaMKII. (E-F) IF quantification analysis of GPX4 and SLC7A11. (G) Quantification analysis of number of vessels in wound. (H) Quantification analysis of blood flow volume on day 3 and day 7. ^*^ indicates a comparison between the two groups. ^*^p <0.05; ^**^p < 0.01; ^***^p < 0.001. All data are from n ≥ 3 independent experiments.


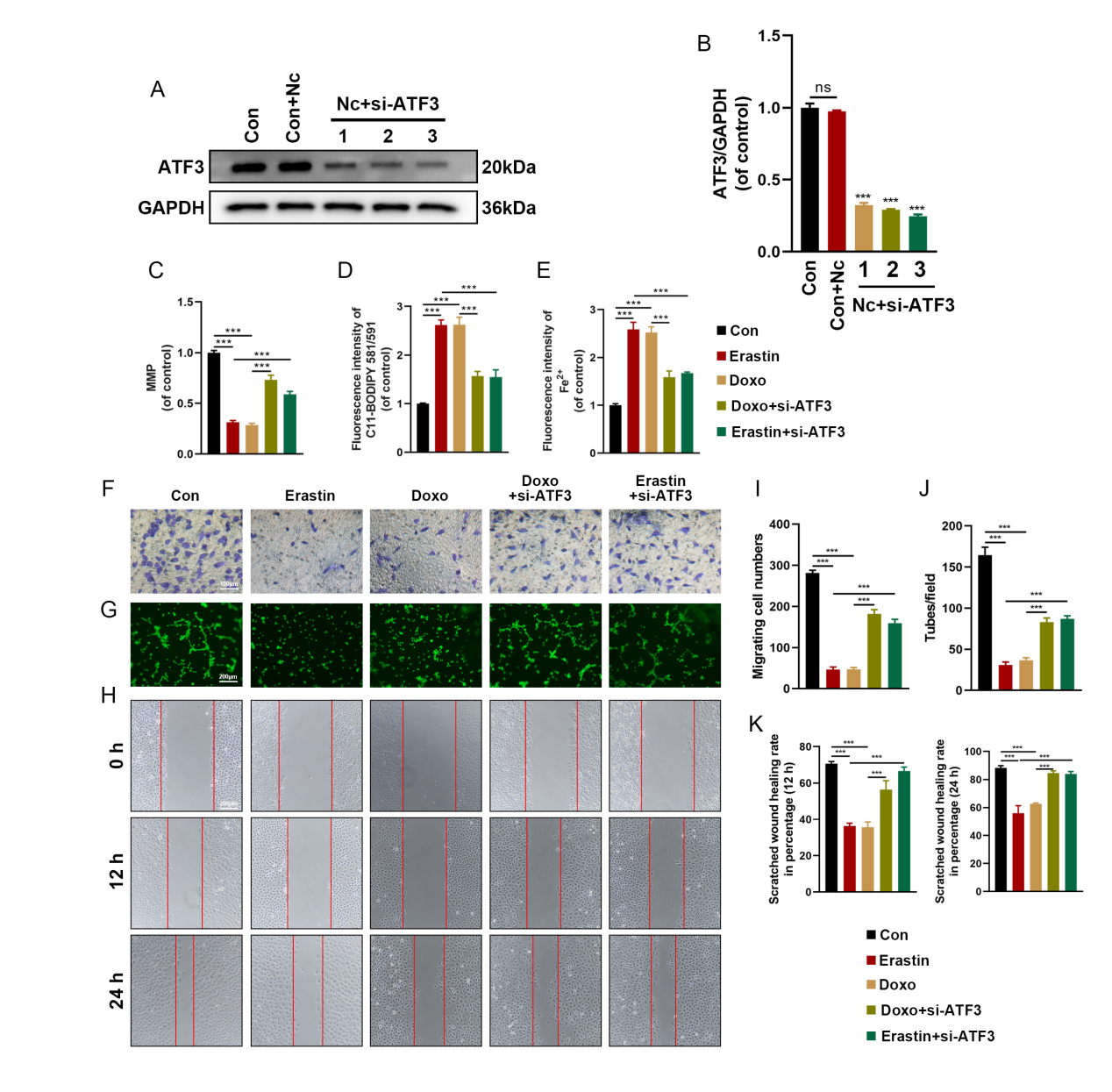


**SFig. 8 Knockdown of ATF3 inhibited ferroptosis and restored the function of senescent HUVECs.** (A-B) Western blot analysis for ATF3 with different treatments. (C) Quantification analysis of the MMP. (D) Quantification analysis of the fluorescence intensity of C11-BODIPY. (E) Quantification analysis of the fluorescence intensity of Fe^2+^. (F-H) Representative images of migration assay, tube formation assay and scratch experiment in HUVECs with different treatments. (I) Quantification analysis of migrating cell numbers. (J) Quantification of tube-forming ability. (K) Quantification of the scratch migration assay. ^*^ indicates a comparison between the two groups. ^***^p < 0.001, ns = no significant. All data are from n ≥ 3 independent experiments.

**
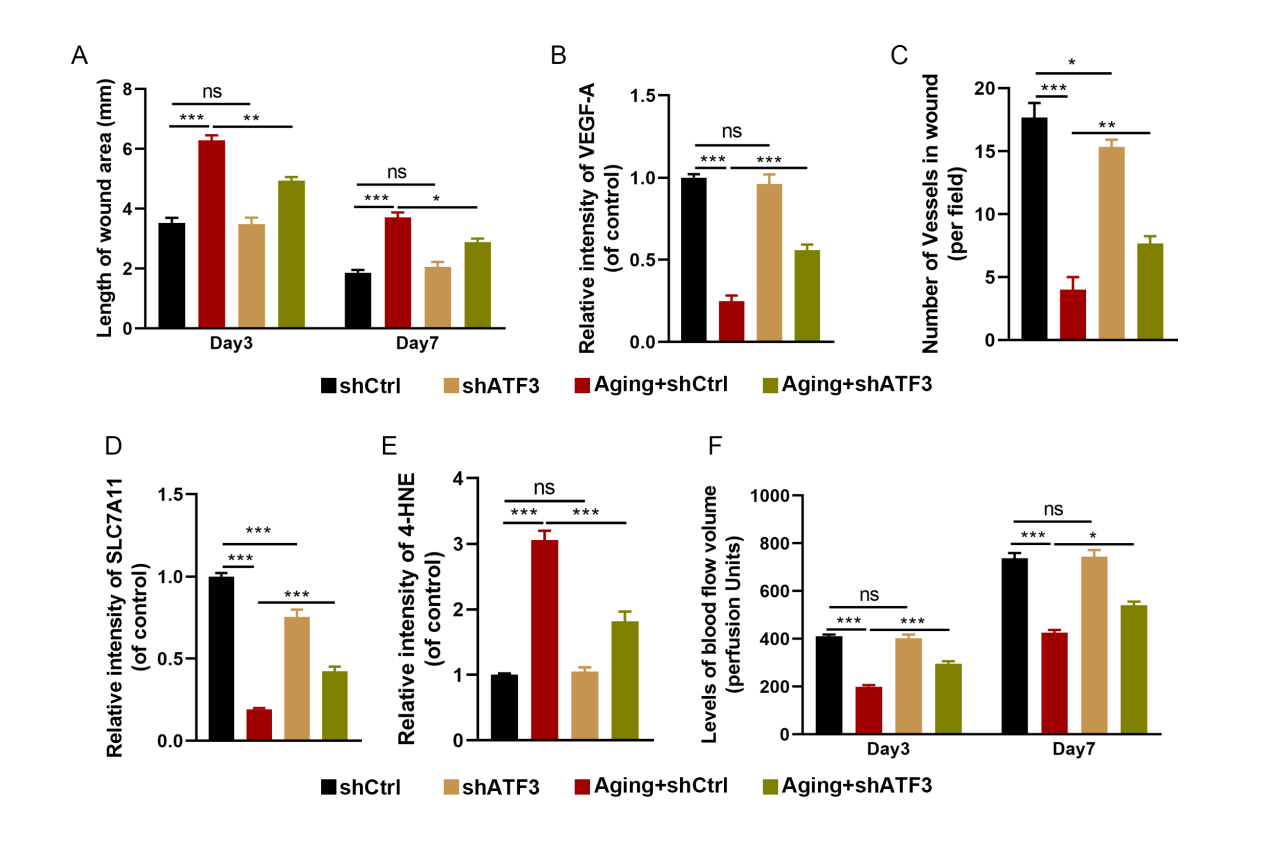
**

**SFig. 9 AAV-ATF3 injection attenuates ferroptosis, promotes aging wound healing and angiogenic.** (A) Quantification analysis of length of wound are in different groups. (B) IHC quantification analysis of VEGF-A. (C) Quantification analysis of number of vessels in wound. (D) IF quantification analysis of SLC7A11. (E) IF quantification analysis of 4-HNE. (F) Quantification analysis of blood flow volume on day 3 and day 7. ^*^ indicates a comparison between the two groups. ^*^p <0.05; ^**^p < 0.01; ^***^p < 0.001; ns = no significant. All data are from n ≥ 3 independent experiments.

**Supplementary Table S1. Primers used for si-Piezo1 and si-ATF3**

| Gene name | Species |  |
| --- | --- | --- |
| piezo1- sense(5-3) | Human | AGAAGAAGAUCGUCAAGUA |
| piezo1- antisense(5-3) | Human | UACUUGACGAUCUUCUUCU |
| ATF3-sense(5-3) | Human | GAAGAAGAUGAAAGGAAAATT |
| ATF3- antisense(5-3) | Human | UUUUCCUUUCAUCUUCUUCTT |

**Supplementary Table S2. Primers used for Gene identification**

| Gene name | Species |  |
| --- | --- | --- |
| piezo1^-/ -^ - Forward | Mouse | CCAGTGATTCCTCATGGAATGTGG |
| piezo1^-/ -^ - Reverse | Mouse | CTTAAGCCCATCTCACAGCTGAAGC |
| Cdh5 3’- Forward | Mouse | ATCCGTGGAGGAGACGGACCAAA |
| Cdh5 3’- Reverse | Mouse | CAACCTTTGTTCATGGCAGCCAGC |

**Supplementary Table S3. The Primary Antibodies**

| Antibody | Isotype | Manufacturer | Cat.No | Dilution |
| --- | --- | --- | --- | --- |
| Piezo1 | Rabbit | Proteintech | 15939-1-AP | 1:1000 |
| ACSL4 | Rabbit | Proteintech | 22401-1-AP | 1:1000 |
| GPX4 | Mouse | Proteintech | 67763-1-Ig | 1:1000 |
| 4-HNE | Rabbit | abcam | Ab46545 | 1:1000 |
| SLC7A11 | Rabbit | Cell Signaling Technology | #98051 | 1:1000 |
| FTH | Rabbit | Cell Signaling Technology | #3998 | 1:1000 |
| CD31 | Mouse | abcam | Ab222783 | 1:1000 |
| VEGF-A | Mouse | Proteintech | 19003-1-AP | 1:1000 |
| CaMKII | Mouse | abcam | Ab134041 | 1:1000 |
| ATF3 | Rabbit | Cell Signaling Technology | #18665 | 1:1000 |
| α-SMA | Mouse | Proteintech | 67735-1-Ig | 1:500 |
| GAPDH | Rabbit | Cell Signaling Technology | #2118 | 1:4000 |
| β-actin | Rabbit | Cell Signaling Technology | #8457 | 1:2000 |
| Lamin B | Rabbit | Cell Signaling Technology | #13435 | 1:1000 |
